# The tyrosine phosphorylated pro-survival form of Fas intensifies the EGF-induced signal in colorectal cancer cells through the nuclear EGFR/STAT3-mediated pathway

**DOI:** 10.1038/s41598-018-30804-z

**Published:** 2018-08-20

**Authors:** Ngoc Ly Ta, Krittalak Chakrabandhu, Sébastien Huault, Anne-Odile Hueber

**Affiliations:** 1 Université Côte d’Azur, CNRS, Inserm, iBV, Côte d’Azur, France; 2 The University of Da-Nang, University of Science and Technology, Da-Nang, Vietnam

## Abstract

Tyrosine phosphorylation of Fas (TNFRSF6/CD95) in its death domain turns off Fas-mediated apoptosis, turns on the pro-survival signal, and has implications in different cancers types. We show here that Fas in its pro-survival state, phosphorylated at Y291 (pY291-Fas), functionally interacts with the epidermal growth factor receptor (EGFR), a key cancer-driving protein and major therapeutic target. Using an evolution-guided pY291-Fas proxy, RNA interference, and site-specific phospho-protein detection, we show that pY291-Fas significantly intensifies EGFR signaling in anti-EGFR-resistant colorectal cancer cells via the Yes-1/STAT3-mediated pathway. The pY291-Fas is essential for the EGF-induced formation of the Fas-mediated nuclear EGFR/STAT3 signaling complex consisting of Fas, EGFR, Yes-1, Src, and STAT3. The pY291-Fas accumulates in the nucleus upon EGF treatment and promotes the nuclear localization of phospho-EGFR and phospho-STAT3, the expression of cyclin D1, the activation of STAT3-mediated Akt and MAPK pathways, and cell proliferation and migration. This novel cancer-promoting function of phosphorylated Fas in the nuclear EGFR signaling constitutes the foundation for developing pro-survival-Fas targeted anti-cancer therapies to overcome disease recurrence in patients with anti-EGFR resistant cancer.

## Introduction

Fas (TNFRSF6/CD95), a member of the tumor necrosis factor receptor superfamily, can either induce apoptosis, which is essential for shutting down chronic immune responses^1–3^ and preventing autoimmunity and cancer^4^, or mediate cell survival, proliferation, and motility, which can promote autoimmunity, cancer growth, and metastasis^5–10^. With increasing evidence of Fas-mediated pro-survival signaling, the cancer-promoting activities of Fas are now recognized as significant and clinically relevant^11^. While inhibiting these activities has shown some clinical promise^12^, the full benefit of this strategy will require a better understanding of the Fas-mediated non-apoptosis signaling.

Recently, we have demonstrated that phosphorylation of Fas at tyrosines 232 and 291 (Y232 and Y291) in its intracellular death domain, is a reversible anti-apoptotic/pro-survival multi-signaling switch that determines the outcome of Fas signaling^13^. The tyrosine phosphorylation turns off the proapoptotic signal and turns on the pro-survival signals that lead to colorectal cancer cell proliferation and migration induced by its ligand, Fas ligand (FasL/TNFSF6/CD95L). Furthermore, we reported elevated levels of Fas death domain tyrosine phosphorylation, which were a direct molecular indicator of Fas pro-survival signal output, in malignant tissues from some cancer types such as colon, breast, and ovarian cancers^13^. These data suggest the probability that the pro-survival signal of Fas may dominantly operate in these cancers.

To date, little is known within the complex pro-survival signaling network in cancer regarding the crosstalk between Fas signaling and other cancer-promoting pathways. The epidermal growth factor receptor (EGFR/HER1/ErbB1) is one of the key cancer-driving proteins and an important target of several anti-cancer therapies^14^. However, a significant number of patients with *KRAS* gene mutations do not positively respond to EGFR-targeting agents such as cetuximab, panitumumab, and erlotinib. And for those who appear to have the *KRAS* wild-type gene and benefit from these drugs initially, resistance inevitably arises and results in a gain in the median progression-free of only less than 1 year^15^. This situation necessitates the investigation into the mechanism of drug resistance and the search for predictive biomarkers and other molecular targets for more adapted combinatory targeted therapies.

As the newly-appreciated Fas survival signaling is a significant contributor to cancer cell survival and aggressiveness^5,16^, we turn our focus toward the relationship between Fas non-apoptotic signaling and EGFR signaling in cancer. Activation of EGFR by its ligands such as the epidermal growth factor (EGF), TGFα, and amphiregulin results in the receptor dimerization and, subsequently, autophosphorylation of a series of tyrosines in the C-terminal tail of the receptor which can influence different cellular effects including proliferation, migration, differentiation, and apoptosis^17,18^. The ras/raf/MEK/ERK, PI3K-Akt, and JAK-STAT are among the pathways classically activated by EGFR. Additionally, a novel signaling pathway influenced by the non-canonical nuclear EGFR signal has emerged^19^. To date, only one report has suggested a strong impact of Fas survival signaling on EGFR pathway in cancer based on the observation that the downregulation of Fas pathway through RNA interference conferred the dependence of lung cancer cells on mutant EGFR oncogene, increasing their sensitivity to the EGFR tyrosine kinase inhibitor, erlotinib^20^. Since then, there has been little progress in understanding the influence of Fas signaling on the EGFR pathway in cancer. Here we report that the pro-survival form of Fas not only crosstalks with the EGFR but also significantly intensifies EGFR signaling in anti-EGFR-resistant colorectal cancer cells via the Yes-1/STAT3-mediated pathway. Fas death domain phosphorylation, which switches on the prosurvival signal of Fas, is essential for the EGF-induced formation of a complex consisting of Fas, EGFR, Yes-1, Src, and STAT3. The phosphorylated Fas (pY291-Fas) accumulates in the nucleus upon cell activation with EGF and promotes the nuclear localization of phospho-EGFR and phospho-STAT3, the expression of cyclin D1, the activation of STAT3-mediated Akt and MAPK pathways, and proliferation and migration of these cells. This report presents the first description of how Fas survival mode intensifies the non-canonical EGFR signals in cancer cells, which have been implicated in worse prognosis and drug resistance in various cancer types^21–23^. This information can be the basis for applying an anti-Fas therapy to overcome recurrence and prolong disease remission of patients with primary and secondary resistance to EGFR targeted therapies.

## Results

### The prosurvival pathway of Fas is upregulated upon the EGF-induced activation of EGFR

**Figure 1:**
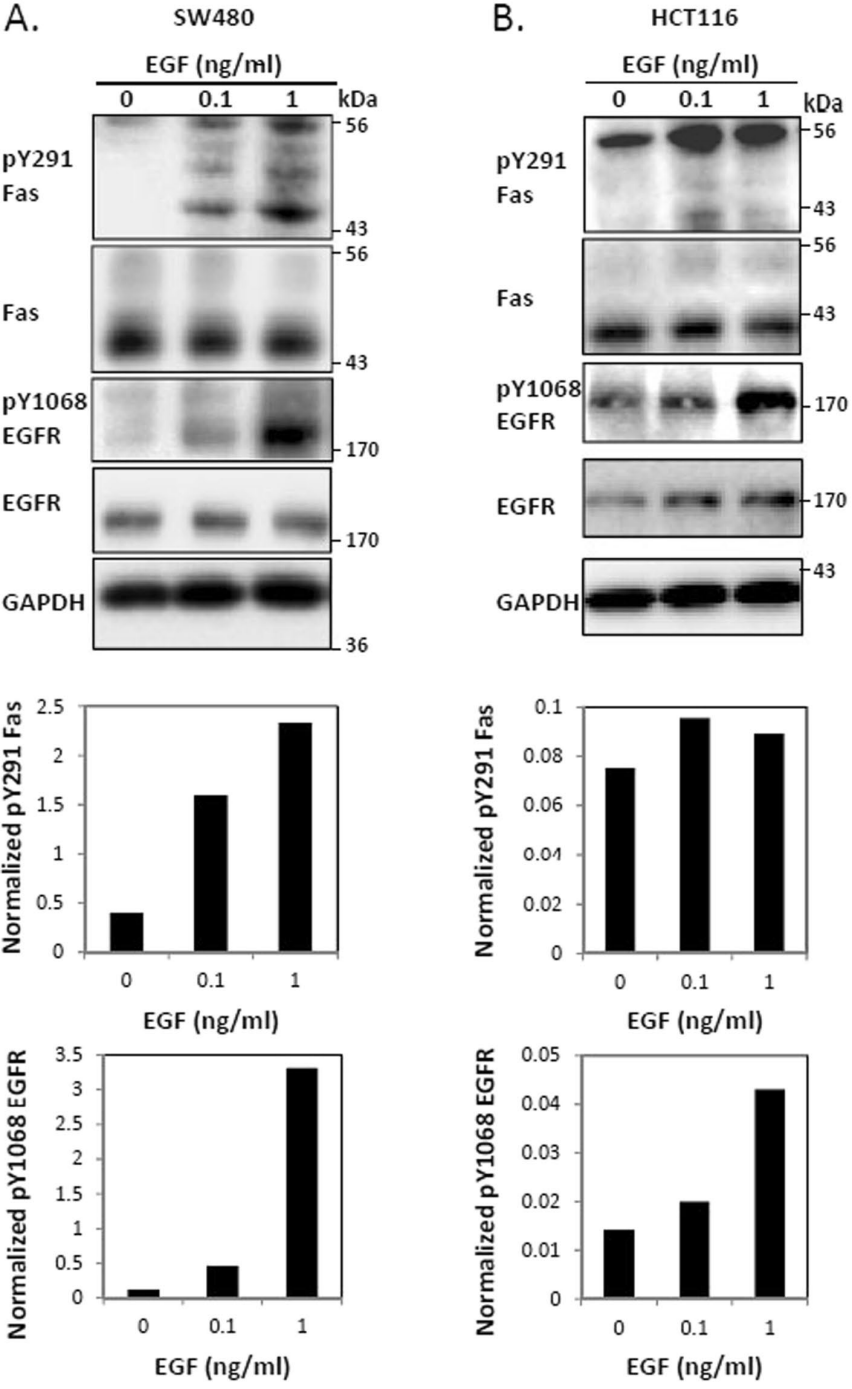
Fas is activated by EGF-induced EGFR signaling. SW480 (**A**) and HCT116 (**B**) cells were incubated with or without indicated concentrations of EGF (ng/ml) for 5 minutes before cell lysates were collected and subjected to SDS-PAGE and immunoblotting with indicated antibodies. Graphs in the lower panel of (**A**,**B**) present the quantification of pY291 Fas or pY1068 EGFR from the corresponding immunoblots normalized with the level of GAPDH. Data are representative of 3 independent experiments.

We have recently reported that Fas activation by sub-lethal doses of soluble FasL leads to the tyrosine phosphorylation of the Fas death domain, which turns on Fas-mediated survival signaling, leading to cell proliferation and migration^13^. To analyze the influence of Fas survival pathways on the EGFR signaling in cancer, we examined the EGF-induced activation of EGFR in two *KRAS*-mutated, cetuximab-resistant colorectal cancer cells, SW480 and HCT116. We found that EGF-induced EGFR activation significantly activated Fas survival signals, as indicated by the increase in the level of phosphorylated Y291 (pY291) Fas upon EGF treatment (Figs 1A,B, and S1). These observations suggest the involvement of pY291-Fas in the survival signaling crosstalk between Fas and EGFR in colorectal cancer cells.

### Fas is essential for the EGFR activation in colorectal cancer cells

**Figure 2:**
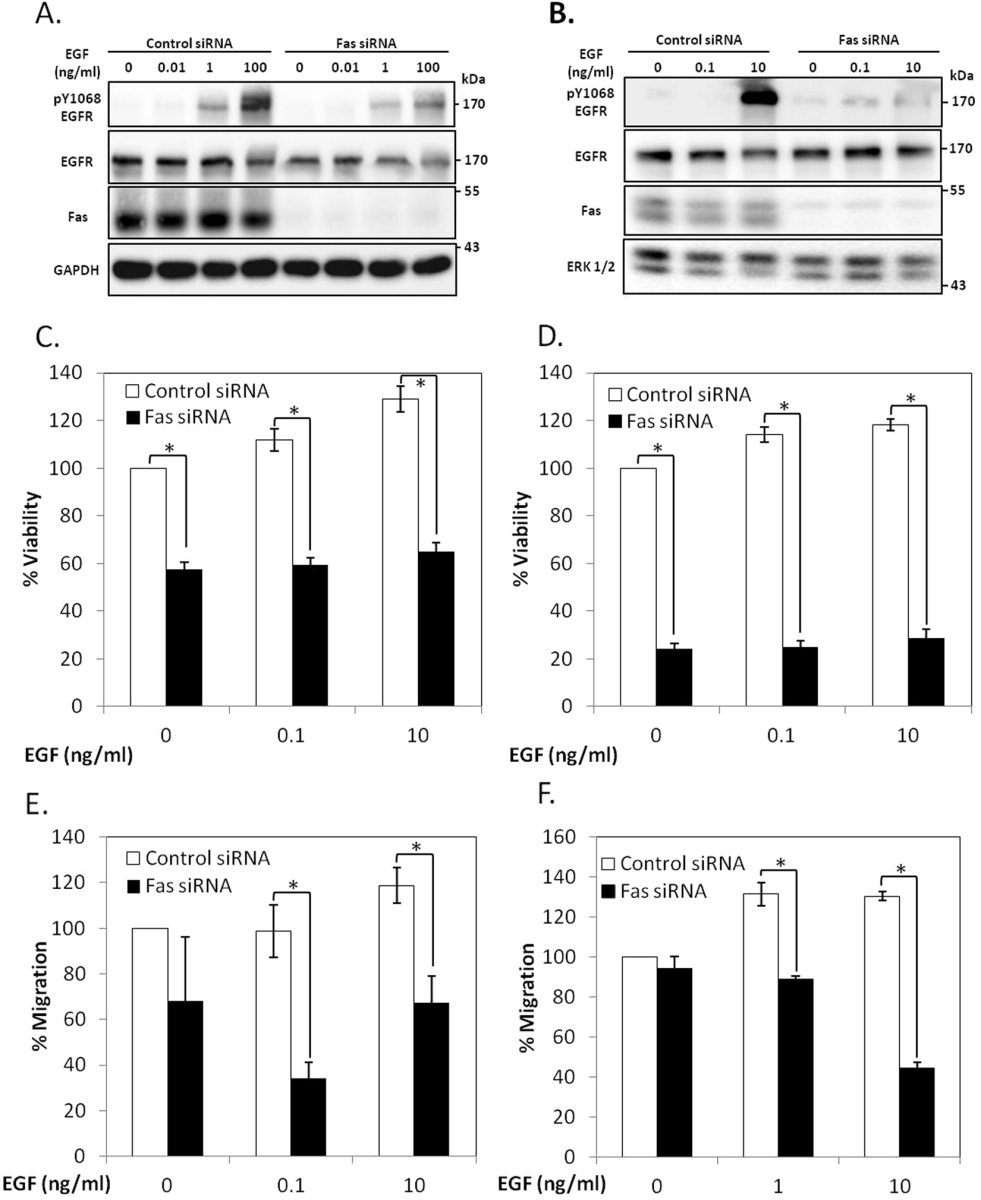
Fas signaling is essential for EGFR activation in colorectal cancer cells. SW480 (**A**) and HCT116 (**B**) cells were transfected with control or Fas siRNA for 48 h. They were subsequently synchronized to G1 phase by serum deprivation for 24 h and then treated with indicated concentrations of EGF (ng/ml) for 5 minutes before cell lysates were collected and subjected to SDS-PAGE and immunoblotting with indicated antibodies. Data are representative of 3 independent experiments. SW480 (**C**) and HCT116 (**D**) cells were transfected with control or Fas siRNA, synchronized as in (**A**,**B**). They were then treated with EGF before analyzing for cell viability by the WST-1 method. Percent viability compared to cells transfected with control siRNA without EGF treatment is presented. SW480 (**E**) and HCT116 (**F**) cells were transfected with control or Fas siRNA, synchronized as in (**A**,**B**). They were then subjected to EGF-induced chemotaxis migration test by Boyden chamber assay. Control cells were allowed to migrate through the chamber without induction with EGF. Percent of cells migrating through the chamber compared to cells transfected with control siRNA without EGF treatment is shown. Means ± SEM of 3 independent experiments are shown (*P < 0.05, unpaired t-test).

We then investigated the importance of the Fas receptor in the EGFR signaling using an siRNA knockdown approach. We found that SW480 and HCT116 cells transiently transfected with a Fas siRNA displayed a significant reduction of the EGF-induced activation of EGFR (Fig. 2A,B) and consequently, an inhibition of both EGF-induced cell proliferation, based on the WST-1 viability assay (Fig. 2C,D), and the EGF-driven directional migration in the Boyden chamber assay (Fig. 2E,F). These data support the notion that Fas significantly contributes to the pro-survival signaling of EGFR in colorectal cancer cells.

### Activation of Fas pro-survival pathway by death domain tyrosine phosphorylation upregulates the EGFR signaling

**Figure 3:**
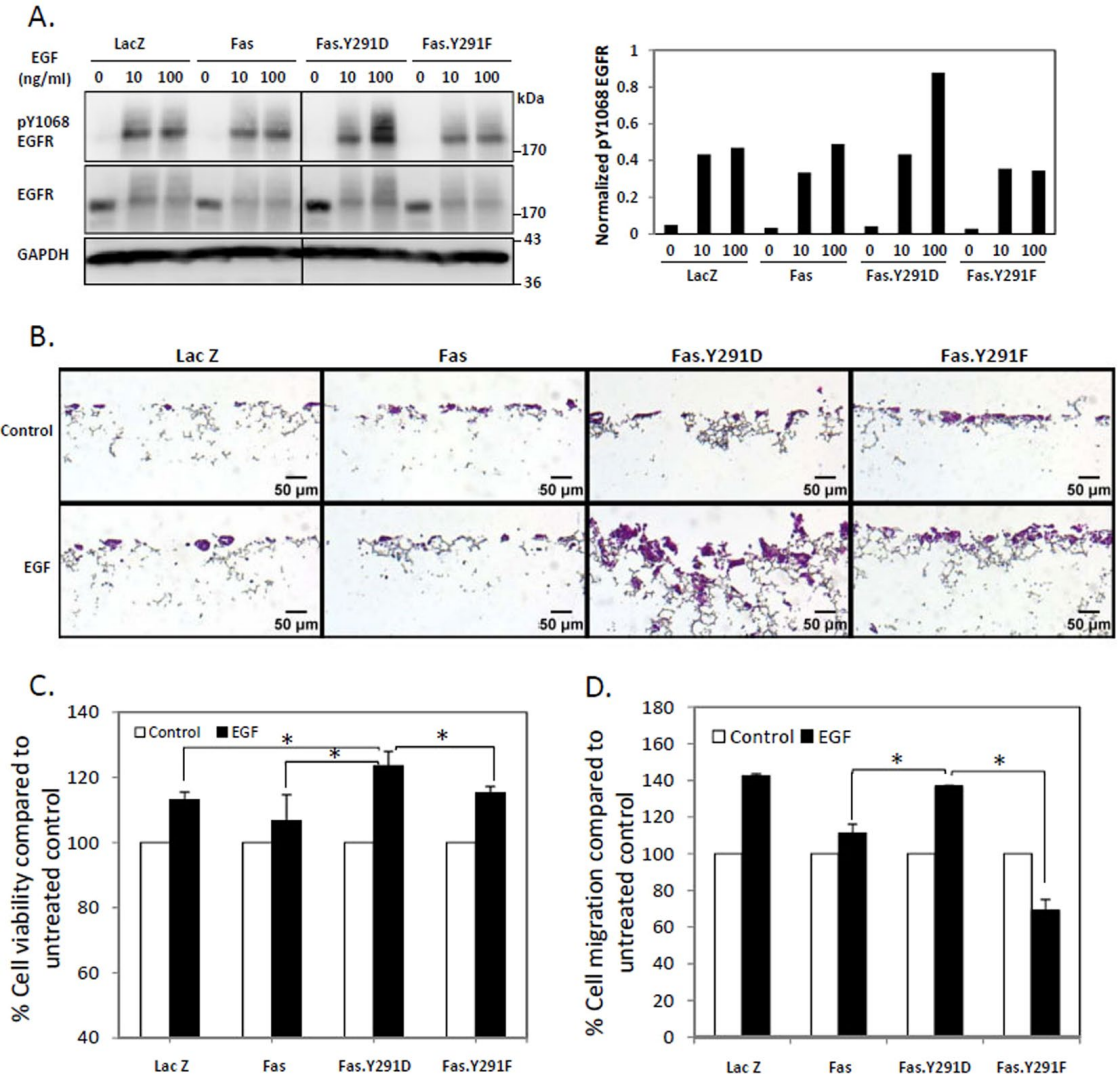
Activation of Fas pro-survival pathway by Fas death domain tyrosine phosphorylation upregulates EGFR signals. (**A**) SW480 cell lines (Fig. S3) stably expressing control protein (LacZ.V5), V5-tagged wild-type Fas, the pY291 Fas proxy (Fas.Y291D), or unphosphorylated Y291 Fas (Fas.Y291F) were synchronized for 24 h then treated with indicated doses of EGF for 5 minutes before cell lysates were collected and subjected to SDS-PAGE and immunoblotting with indicated antibodies. The plot in the right panel presents the quantification of pY1068 EGFR from the immunoblot normalized with the level of GAPDH. Data are representative of 2 independent experiments (**B**) SW480 cells as in (**A**) were grown in 3-dimensional cell culture scaffold (Alvetex) coated with matrigel for 14 days without or with 0.01 ng/ml EGF, then fixed and stained with hematoxylin/eosin. Images were taken with 10x magnification. (**C**) SW480 cells as in (**A**) were synchronized for 24 h, then treated with 100 ng/ml EGF for 48 h before analyzing for cell viability by the WST-1 method. Percent viability compared to untreated cells is presented. (**D**) SW480 cells as in (**A**) were synchronized for 24 h, then subjected to EGF-induced chemotaxis migration test by Boyden chamber assay. Control cells for each cell line were allowed to migrate through the chamber without induction with EGF. Percent of cells migrating through the chamber compared to control cells is presented. Means ± SEM of 3 independent experiments are shown (*P < 0.05, unpaired t-test).

While Fas siRNA experiments suggested the importance of Fas for an efficient EGFR signaling, it remained unclear whether the activation of the pro-survival mode of Fas was required for its role as a positive regulator of EGFR signaling. To answer this question, we made use of a proxy for Fas death domain phosphotyrosine (pY291), i.e., the Y291D mutation, which we had verified through our previous evolution-guided analysis as a functional substitute for pY291 that maintained Fas in the pro-survival mode^13^. We found that the pY291 Fas mimetic (Fas.Y291D) enhanced the EGFR signaling triggered by two of its ligands, EGF (Figs 3A, S3A) and amphiregulin (Fig. S3B). This effect was not observed with the unphosphorylated Y291 Fas mimetic (Fas.Y291F), which was a stand-in for the constitutively pro-apoptotic form of Fas^13^. In accordance with their increased EGFR activation, when compared to either control cells or cells carrying wild-type Fas or Fas.Y291F, cells carrying the Fas.Y291D exhibited enhanced EGF-triggered cell growth and penetration through 3D culture scaffolds, which enables cells to maintain their morphology, behavior, and responsiveness more closely to the *in vivo* state (Fig. 3B). They also exhibited an increased EGF-induced cell proliferation based on WST-1 viability assay (Fig. 3C) and directional cell migration toward EGF in Boyden chamber assay (Fig. 3D). Thus, the phosphorylation of Fas at the Y291 in the death domain, which turns Fas into the pro-survival state, is essential for its role in intensifying EGFR survival signals.

### The pro-survival form of Fas is the signaling hub required for EGF-induced formation of protein complexes consisting of Fas, EGFR, Src, Yes-1, and STAT3

**Figure 4:**
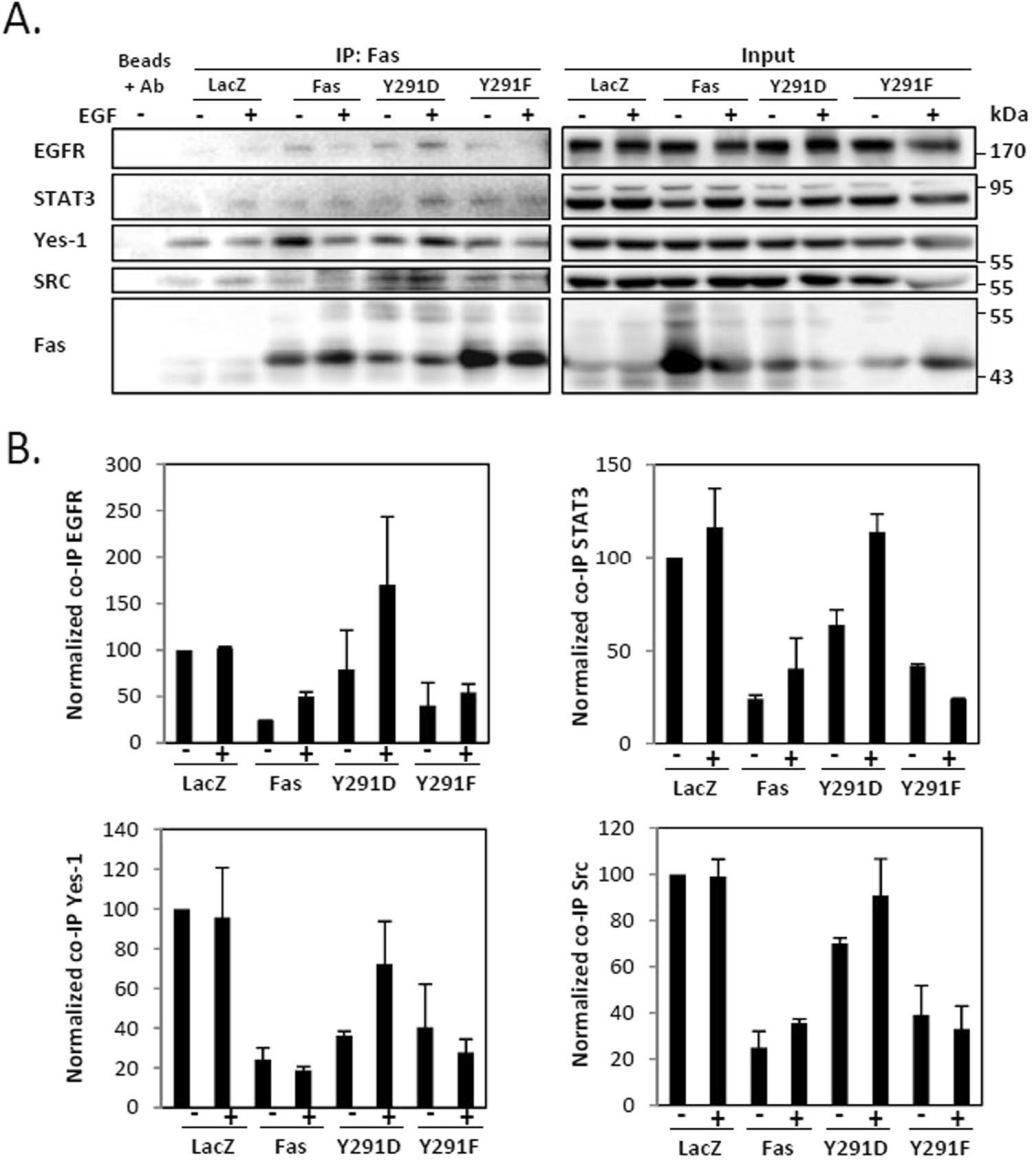
The pro-survival form of Fas promotes the EGF-induced formation of protein complexes comprising Fas, EGFR, Src, Yes-1, and STAT3. (**A**) SW480 cell lines stably expressing indicated proteins were synchronized for 24 h then incubated without or with 10 ng/ml EGF for 5 minutes. Cell lysates were collected and Fas co-immunoprecipitation was performed, followed by SDS-PAGE and immunoblotting with indicated antibodies. Data are representative of 2 independent experiments. (**B**) Quantification of the level of indicated protein co-immunoprecipitated with Fas from cells treated as in (**A**). The level of each co-immunoprecipitated protein was normalized with the level of immunoprecipitated Fas. Means ± SEM of 2 independent experiments are shown.

All the knowledge we currently have about Fas-EGFR crosstalk comes only from the standpoint that considers the contribution of EGFR in FasL-induced signaling. Previous work has suggested that through the Src-family kinase, Yes-1, EGFR amplifies FasL-induced cell migration in triple-negative breast cancer cells^24^. Meanwhile, the connection between Fas and EGFR through which the pro-survival mode of Fas heightens the EGF-induced EGFR signaling in cancer was never addressed. By means of co-immunoprecipitation, we found that upon the activation with EGF, the pro-survival form of Fas (Fas.pY291D) promoted a rapid formation of complexes that included Fas, EGFR, the Src-family kinases, Src and Yes-1, and STAT3 (Fig. 4). This complex formation was inhibited when Fas was in a pro-apoptotic form (Fas.Y291F) (Fig. 4). This observation indicates the requirement of the prosurvival form of Fas for the formation of a signaling platform where Src, Yes-1, and STAT3 may couple the pro-survival signal of Fas to EGF-induced EGFR signaling.

### Yes-1 and Src play opposing regulatory roles in STAT3 activation in the Fas-mediated EGFR pathway

**Figure 5:**
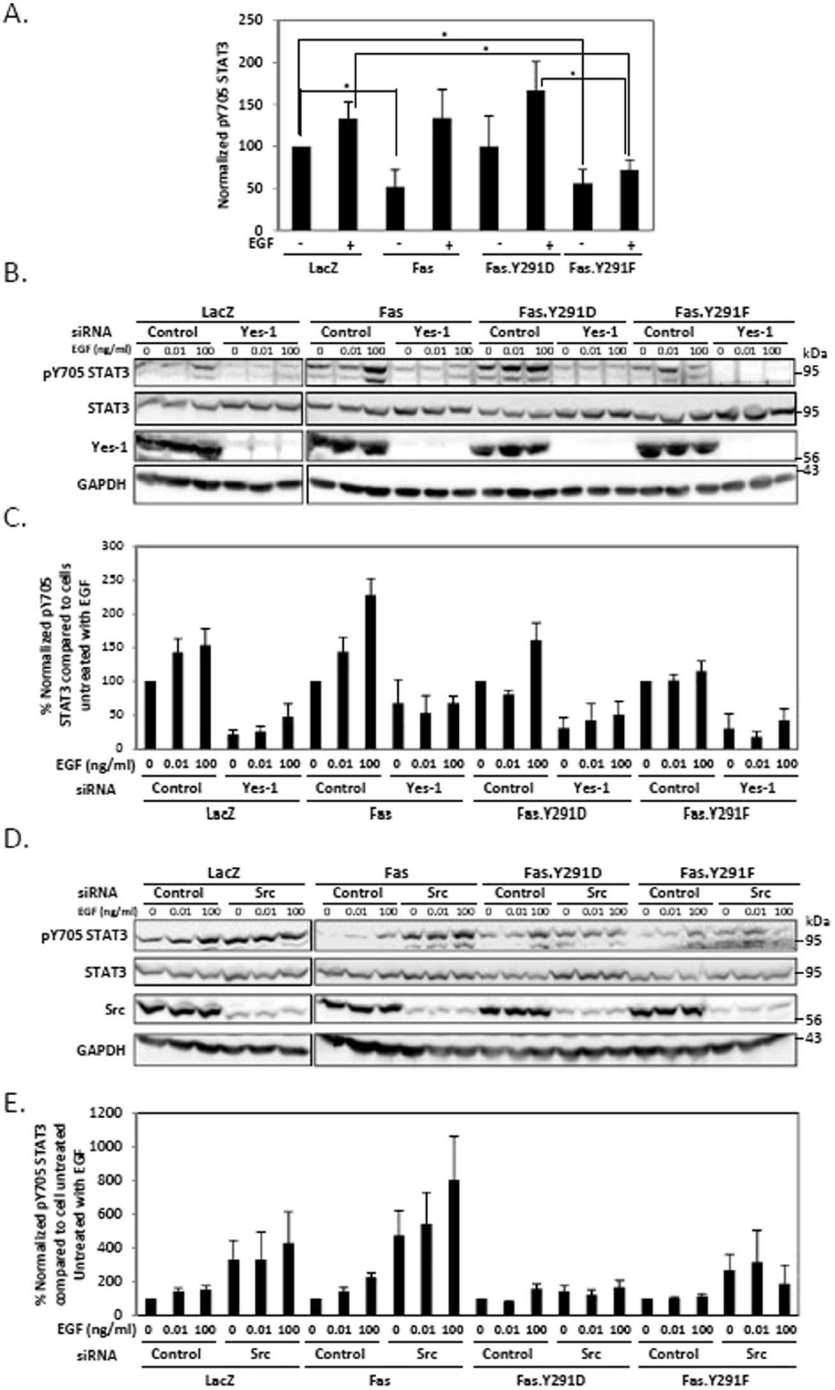
Yes-1 and Src play opposite roles in the regulation of STAT3. (**A**) Quantification of pY705 STAT3 of SW480 cell lines stably expressing indicated proteins. Cells were synchronized to G1 phase by serum deprivation for 24 h and then treated with 100 ng/ml of EGF for 5 minutes before cell lysates were collected and subjected to SDS-PAGE and immunoblotting with indicated antibodies. The level of pY705 STAT3 from immunoblots was normalized against the level of GAPDH. Means ± SEM of 4 independent experiments are shown (*P < 0.05, unpaired t-test). (**B**) Cells as in (**A**) were transfected with control or Yes-1 siRNA for 48 h then synchronized as in (**A**) and treated with indicated concentrations of EGF (ng/ml) for 5 minutes before cell lysates were collected and subjected to SDS-PAGE and immunoblotting with indicated antibodies. Data are representative of 3 independent experiments. (**C**) Quantification of pY705 STAT3 of cells treated as in (**B**). The level of pY705 STAT3 from immunoblots was normalized against the level of GAPDH. Means ± SEM of 3 independent experiments are shown. (**D**) Cells as in (**A**) were transfected with control or Src siRNA for 48 h then synchronized as in (**A**) and treated with indicated concentrations of EGF (ng/ml) for 5 minutes before cell lysates were collected and subjected to SDS-PAGE and immunoblotting with indicated antibodies. Data are representative of 4 independent experiments. (**E**) Quantification of pY705 STAT3 of cells treated as in (**D**). The level of pY705 STAT3 from immunoblots was normalized against the level of GAPDH. Means ± SEM of 4 independent experiments are shown.

STAT3 is one of the key downstream effector signaling cascades regulated by Src family kinases (SFKs), a family of nine non-receptor protein tyrosine kinases including Src and Yes-1^25^. The activation of STAT3 by Src is well characterized^26^ while the role of Yes-1 in STAT3 activation is unknown. In terms of Fas prosurvival signaling, we previously demonstrated that both Src and Yes-1 positively regulated the phosphorylation of Fas interchangeably^13^. Since Src and Yes-1 were present along with STAT3 in the Fas.Y291D-mediated protein complex (Fig. 4), we wished to discern the respective roles of Src and Yes-1 in the EGF-induced STAT3 activation. We found that, upon SW480 cell activation with EGF, the prosurvival form of Fas (Fas.Y291D) promoted the phosphorylation of STAT3 at Y705 residue while the proapoptotic form of Fas (Fas.Y291F) inhibited it (Fig. 5A). Furthermore, the suppression of Yes-1 expression by siRNA abolished phospho-705 (pY705) STAT3, indicating that Yes-1 was indispensable for STAT3 activation (Fig. 5B,C). On the other hand, and contrary to the commonly reported role of Src as a STAT3 activator, we observed no reduction, but rather an amplification of STAT3 activation upon Src suppression by siRNA (Fig. 5D,E). The upregulation of pY705 STAT3 by the inhibition of Src by siRNA was observed in all the cells tested. However, the upregulation of pY705 STAT3 by Src siRNA in Y291D and Y291F carrying cells was less striking when compared to the effect in Fas wild type-carrying cells. Nonetheless, our data not only demonstrate the important role of the prosurvival Fas as a mediator of EGF-induced STAT3 activation but also underline a novel evidence of selective activities of different SFKs in colorectal cancer cells where Src and Yes-1 play opposite roles in the regulation of STAT3 activity.

### The pro-survival form of Fas promotes nuclear EGFR/STAT3 signaling

**Figure 6:**
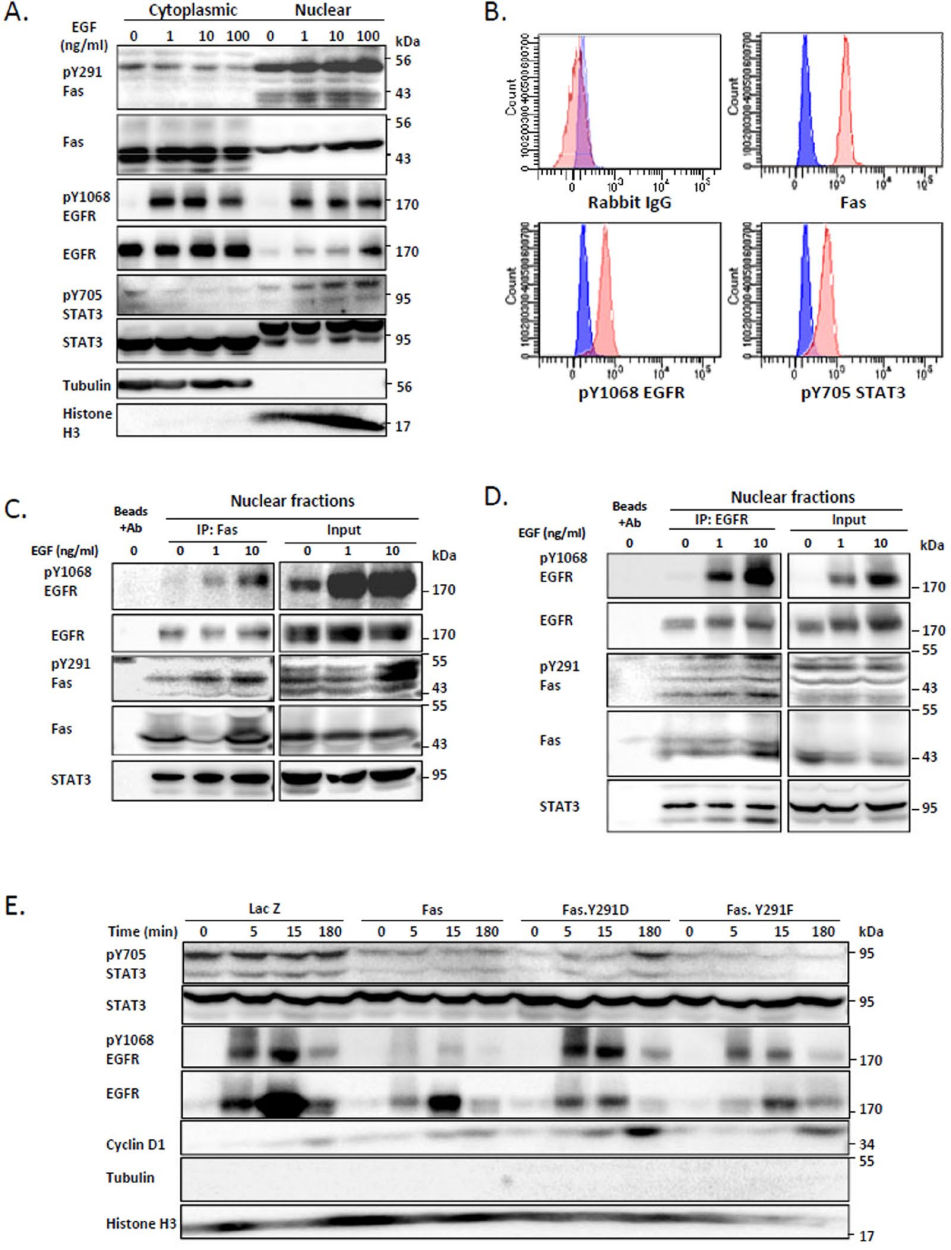
The phosphorylated Fas is required for EGF-induced nuclear EGFR/STAT3 signaling. (**A**) SW480 cells were synchronized for 24 h before being treated with the indicated concentrations of EGF (ng/ml) for 3 h. They were then subjected to nuclear fractionation followed by SDS-PAGE and immunoblotting with indicated antibodies. Data are representative of 3 independent experiments. (**B**) The nuclear fraction of SW480 cells were subjected to IP-FCM and the levels of indicated proteins co-immunoprecipitated with Fas were analyzed by flow cytometry. Blue, IP beads incubated with Alexa Fluor 647 conjugated secondary antibody; red, IP beads incubated with an unrelated IgG or an antibody directed against indicated protein followed by a corresponding secondary antibody. Data are representative of 2 independent experiments. (**C**) Synchronized SW480 cells overexpressing Fas were treated with the indicated concentrations of EGF (ng/ml) for 15 minutes and subsequently subjected to nuclear fractionation. Fas co-immunoprecipitation was then performed on the nuclear fractions followed by immunoblotting with indicated antibodies. Data are representative of 2 independent experiments. (**D**) Synchronized SW480 cells were treated as in (**C**). EGFR co-immunoprecipitation was then performed on the nuclear fractions followed by immunoblotting with indicated antibodies. Data are representative of 2 independent experiments. (**E**) SW480 cell lines stably expressing indicated proteins were synchronized as in (**A**) then treated with 10 ng/ml EGF for the indicated period of time. They were then subjected to the nuclear fractionation, SDS-PAGE, and immunoblotting.

The critical role of Yes-1^27^ and STAT3^28^ in the translocation of EGFR to the nucleus, an event correlated with poor survival in several types of cancers^21,22^ and resistance to various anti-cancer therapies^23^, is known. In addition, nuclear EGFR has been shown to associate with STAT3^28^ and cyclin D1 promoter, leading to its transcriptional activation^29^. Since we demonstrated the requirement for the pro-survival form of Fas as a hub for the recruitment of EGFR, Yes-1, and STAT3, and the Yes-1-dependent STAT3 activation, we wished to further investigate the downstream events mediated by the pro-survival form of Fas with regard to the nuclear EGFR signaling. By examining SW480 cells, we observed an increased level of pY291-Fas, along with the increased levels of pY1608-EGFR and pY705-STAT3, in the nuclear fraction upon cell activation by EGF (Fig. 6A). Furthermore, using immunoprecipitation detected by flow cytometry (IP-FCM) which allow the measurement of the complex formation of proteins in their native forms, we found that pY1068 EGFR and pY705 STAT3 co-immunoprecipitated with Fas from the nuclear fraction of the SW480 cells (Fig. 6B), demonstrating that Fas existed in complex with activated EGFR and STAT3 in the nucleus. Using Fas co-immunoprecipitation detected by immunoblotting, we observed that pY291 Fas associated with activated EGFR and STAT3 and that the pY291 Fas/EGFR/STAT3 complex increased with the activation by EGF (Fig. 6C). Reciprocal EGFR co-immunoprecipitation demonstrated the same result (Fig. 6D). These results suggest the involvement of pY291-Fas in the nuclear EGFR/STAT3 signaling. To verify the contribution of the prosurvival form of Fas in the nuclear EGFR/STAT3 signaling, we made use of the pY291-Fas mimetics (Fas.Y291D). We found that, when compared to the wild-type Fas and the dephosphorylated Fas mimetic (Fas.Y291F), the pro-survival form of Fas enhanced the EGF-induced nuclear localization of phosphorylated EGFR and phosphorylated STAT3 (Fig. 6E) as well as cyclin D1 (Figs 6E and S5). These data suggest that the signaling hub that is mediated by the pro-survival pY291-Fas facilitates the transmission of the EGFR signaling via the non-canonical EGFR nuclear transport.

### STAT3 is the key to the enhanced EGF-induced Akt and Erk1/2 activation in cells that express the pro-survival Fas

**Figure 7:**
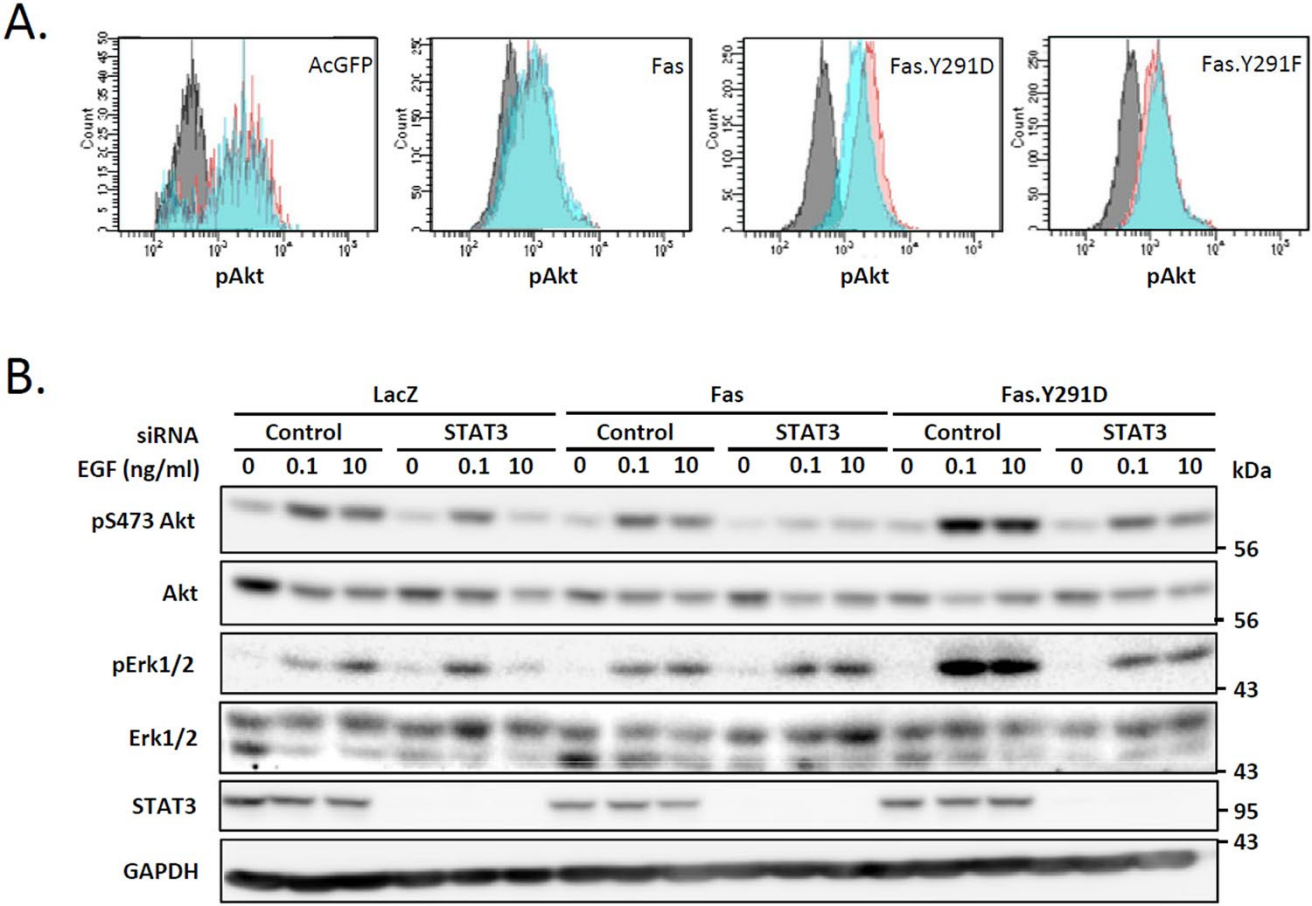
STAT3 is required for the enhanced EGF-induced Akt and Erk1/2 activation in cells that express pro-survival Fas (**A**). SW480 cell lines stably expressing AcGFP or indicated AcGFP-tagged protein were synchronized to the G1 phase by serum deprivation for 24 h and then incubated without (control) or with 10 ng/ml EGF for 5 minutes. The level of pT308 Akt was then analyzed by flow cytometry (Grey, control cells incubated with the secondary antibody; blue and red, control and activated cells, respectively, stained with anti-pAkt followed by fluorescent-conjugated secondary antibody). Data from cells with equivalent levels of AcGFP expression are shown. Data are representative of 2 independent experiments. (**B**) SW480 cell lines stably expressing indicated proteins were transfected with control or STAT3 siRNA for 48 h. They were subsequently synchronized to G1 phase by serum deprivation for 24 h and then treated with the indicated concentrations of EGF (ng/ml) for 5 minutes before cell lysates were collected and subjected to SDS-PAGE and immunoblotting with indicated antibodies. Data are representative of 2 independent experiments.

Along with the STAT3 pathway, the PI3K-Akt and ras/raf/MEK/ERK pathways are well known downstream effectors of EGFR signaling that have been noted as the node of convergence of anti-EGFR drug resistance. Therefore, we examined the effect of the pro-survival form of Fas on these pathways. We found that, compared to cells carrying wild-type Fas and the pro-apoptotic form of Fas (Fas.Y291F), cells carrying the prosurvival Fas.Y291D exhibited a higher level of EGF-induced activation of Akt (Figs 7A,B, and S6A,B) and Erk1/2 (Figs 7B and S6C). Having observed that the pro-survival Fas form was responsible for the enhanced EGF-induced activation of Akt and Erk1/2 whereas the pro-apoptotic Fas exhibited the opposite effect, we further investigate whether STAT3 had a role in the enhanced activation of Akt and Erk1/2 following EGF treatment observed in cells carrying the pro-survival Fas. We found that the Fas-mediated upregulation of Akt and Erk1/2 in these cells was reestablished to the level close to that found in cells carrying the wild-type Fas when STAT3 was suppressed by siRNA (Fig. 7B), indicating the essential role STAT3 in the Fas-mediated pro-survival amplification of EGFR signaling through the PI3K-Akt and ras/raf/MEK/ERK pathways.

## Discussion

More than a decade ago, when Fas was still mostly known and studied as a prototypic apoptosis activator, the Fas-EGFR crosstalk was described in the pro-apoptotic context where hyperosmolarity-induced activation resulted in EGFR activation which in turns promoted Fas-mediated apoptosis in hepatocytes^30^. Later, a study showed that in quiescent hepatic stellate cells, EGFR could participate in FasL-induced proliferative and anti-apoptotic signal^31^. However, since the cancer-promoting roles of Fas have emerged, only a few studies have touched upon the relationship between Fas and EGFR in cancer and mainly focused on the influence of EGFR signaling on Fas pathway and not vice versa^20,24^. Studying triple-negative breast cancer cells, Malleter *et al*.^24^ found that activating Fas with the cleaved form of FasL could promote the migration of these cells via the activation of Yes-1-mediated, EGF-independent EGFR activation and subsequently the activation of the PI3K pathway^24^. Meanwhile, whether Fas has a role in EGF-dependent EGFR activation in cancer remained unknown. Only one work reported by Bivona *et al*.^20^ has suggested an indirect influence of Fas signaling on the EGFR pathway. Activating mutations in EGFR, such as an exon 19 deletion, predict for the lung cancer cell sensitivity to erlotinib, an EGFR tyrosine kinase inhibitor. Using RNAi, the authors showed that Fas could render EGFR mutant lung cancer cells independent of the mutant EGFR and thereby resistant to erlotinib by acting upstream of persistent NFkB signaling and an unidentified MEK-independent pathway^20^. In this case, although, the direct role of Fas in EGFR signaling was not demonstrated, its presence was reported essential for the cancer-promoting activities of other parallel pathways that made the EGFR activity redundant.

Given the correlation between poor prognosis and EGFR overexpression in several types of cancers, anti-EGFR agents have become a major class of targeted therapy^32–34^, albeit, with rarely complete responses and eventual drug resistance in most cases. While a subset of colorectal tumors, apparently wild-type for *KRAS*, are sensitive to EGFR inhibition, they almost always develop secondary resistance after an initial response^35–37^. Recent studies have identified *KRAS* mutations as frequent drivers of acquired resistance to the anti-EGFR therapies, cetuximab and panitumumab^38,39^. These studies also showed that the resistance may arise from the selection of pre-existing *KRAS* mutant clones in metastatic lesion as well as from continuing mutagenesis. Notably, the appearance of *KRAS* mutations in patients whose tumors were initially classified as *KRAS* wild-type occurred consistently between 5–6 months following the anti-EGFR treatment^39^. Therefore, to prolong the remission beyond 5–6 months, the foreseeable resistance must be taken into account and combinatory therapies targeting different pathways or early initiation of sequential therapies targeting other pathways must be applied.

Our recent study has shown that the level of the phosphorylated Fas, which is the form of the receptor that promotes cancer cell proliferation and migration, is elevated in malignant tissues from colorectal, breast, and ovarian cancers^13^. This important observation prompted us to investigate the connection between this pro-survival Fas in EGF-dependent EGFR signaling in colorectal cancer cells, which represents the type of cancer to which anti-EGFR therapy is widely applied. Our finding that the survival signaling pathways of Fas could be promptly activated by EGF, the cognate ligand of the EGFR, signifies a close connection between the two pathways in colorectal cancer cells. In *KRAS* mutant cells used in this study, activating EGFR with EGF led to cell proliferation and migration. This process was strongly inhibited when Fas expression was suppressed by siRNA, demonstrating that perturbing Fas pathway would directly and significantly impact EGF-induced EGFR signaling. Using the pY291 Fas proxy, we found that it was the phosphorylated, pro-survival form of Fas that coupled Fas pathway to the EGFR pathway by acting as the signaling hub for the complex assembly that included EGFR, Src-family kinases, Yes-1 and Src, and STAT3, and permitting a robust EGF-induced EGFR signaling.

Upon activation by EGF, the dimerization of EGFR allows the cross-phosphorylation of several tyrosine residues, including Y1068, the main binding site for STAT3^40^. Our data show that the presence of phosphorylated Fas significantly promoted autophosphorylation of EGFR at Y1068, possibly by facilitating EGFR dimerization, and thus the increased EGFR association with STAT3. Correspondingly, the expression of the pro-survival form of Fas enhanced the EGF-induced phosphorylation of STAT3, which is important for the cancer-promoting activities, such as migration of colorectal cancer cells (Figs S7A and S8). While the exact action of phospho-Fas (pY-Fas) in this process is still unknown, it may involve the membrane dynamics and organization of Fas and EGFR, which may significantly impact their crosstalk. Sequestering signaling partners in membrane domains dramatically increases their encounter while preventing interactions with other interfering proteins. While direct evidence has yet to be put forward, the tyrosine phosphorylation of Fas may be linked to the membrane organization of both Fas and EGFR and thus the heightened EGFR signal. Numerous studies have provided evidence that the activation of EGFR is capable by itself of remodeling its lipid environment, allowing the formation of nanoclusters^41^. The same may also hold true for the activated Fas. Such membrane remodeling may, in turn, influence the sequestration of Fas and EGFR into the same membrane nanodomains, and thus their association (direct or indirect), which may result in an arrangement at the molecular level that favors the recruitment of STAT3 as well as Src and Yes-1.

It is often stated generally that members of the Src family kinases can activate STAT3. Although, while it is quite a common knowledge that Src can activate STAT3^26^, the role of Yes-1 as a STAT3 activator has not yet been demonstrated. Given the known redundancy of SFK activities^13,42^, the presence of both Src and Yes-1 in the Fas-EGFR complex after EGF-induced activation, and the fact that both Src and Yes-1 have been shown as positive regulators of the phosphorylation of Fas^13^ and EGFR^24,43^, one may expect that Src and Yes-1 may interchangeably perform as STAT3 activators. However, in the context of our study, Src and Yes-1 play opposite roles in EGF-induced STAT3 activation, with Yes-1 as the activator and Src as the suppressor of STAT3 phosphorylation at Y705. The presence of both activator and suppressor of STAT3 in the same complex is of interest and the complete functional organization of Src and Yes-1 in the EGF-induced, Fas-mediated signaling complex remains to be investigated.

Interestingly, our data also reveal that while the suppression of Src by siRNA resulted in the upregulation of pY705 STAT3 in all cells tested in this study, this upregulation was less striking in Y291D and Y291F carrying cells than in Fas wild type-carrying cells. It appears that the STAT3 signaling rebound, after the negative regulation by Src was removed, required the reversibility of the Fas tyrosine phosphorylation since a constitutive phosphorylation or dephosphorylation of the Y291 residue reduced this effect. This observation suggests that a well-timed phosphorylation and dephosphorylation of Fas is important for this significant upregulation of STAT3 in the absence of the negative regulation by Src. This is an interesting observation that warrants and further investigation to understand the exact roles of Fas in the Src-mediated negative regulation of STAT3. In any case, the new information we presented regarding opposing roles of two closely related and ubiquitous SFKs in the activation of an emerging drug target such as STAT3 in the pathway of a major clinical drug target like EGFR is highly significant, particularly in consideration of the alternative or combinatory application of SFK inhibitors in anti-cancer therapies.

Classical signaling pathways initiated by EGFR upon engagement by its ligand include key oncogenic pathways such as the ras/raf/MEK/ERK, PI3K-Akt, and JAK-STAT. Non-canonically, EGFR can also be shuttled from the plasma membrane to the nucleus and serves as a transcriptional co-activator for many tumor-promoting genes^19^. Importantly, the nuclear EGFR has also been implicated in the acquired resistance to cetuximab^44^. Our data show that pY291-Fas acted as a hub for the formation of a Fas-mediated EGFR signaling complex which contained Fas, EGFR, Yes-1, and STAT3. Furthermore, it was translocated to the nucleus along with activated EGFR and STAT3 upon activation by EGF. The enhanced nuclear accumulation of pY-Fas, pY-EGFR, and pY-STAT3 was accompanied by the upregulation of cyclin D1, a nuclear EGFR target gene^29^. These findings are in line with previous studies showing that the nuclear translocation of EGFR and the binding and upregulation of its target gene depend on Yes-1^27^ and the association of EGFR with STAT3^28^. Notably, this is the first report of the presence of pY-Fas in the nucleus upon EGF-induced activation, which reinforces the implication of the roles of pY-Fas in nuclear EGFR signaling and the activities of transcription factors. Additionally, we found that, in cells expressing the prosurvival pY-Fas, the increased activation of STAT3 was also responsible for the upregulation of PI3K/Akt. This observation is consistent with previous findings that Akt may be upregulated by STAT3 at the transcriptional level^45^. And while the activation of STAT3 has been shown dependent on MEK/Erk pathway^46,47^, we found that the inverse occurred when the phosphorylated form of Fas was upregulated. Namely, the activation of the MEK/Erk pathway in colorectal cancer cells depended on the activation of STAT3.

**Figure 8:**
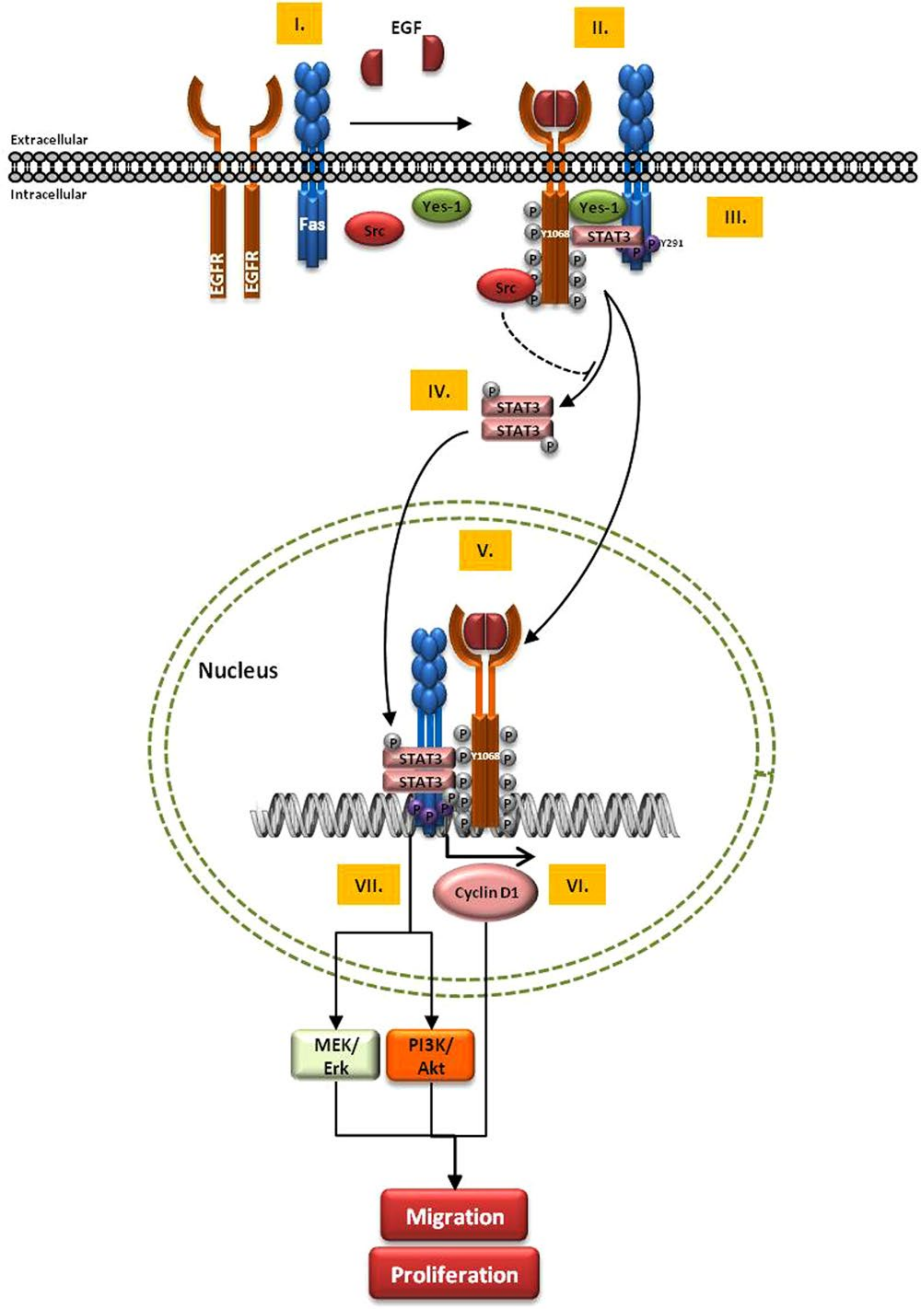
Schematic representation of Fas-mediated EGF-induced EGFR signaling A proposed model of the robust EGF-induced EGFR signaling pathway mediated by the pro-survival, phosphorylated Fas that can lead to aggressive behavior of cancer cells is presented here. I. The presence of Fas is required for the efficient autophosphorylation and thus the activation of EGFR. II. Upon the binding with EGF, the Fas-dependent EGFR activation occurs concomitantly with the activation of the non-apoptotic mode of Fas by death-domain tyrosine phosphorylation at Y291. III. A protein complex comprising EGFR, Fas, Yes-1, STAT3, and Src is formed in a pY291 Fas-dependent manner. IV. STAT3 is then phosphorylated by Yes-1. V. pY-Fas, pY-EGFR, and pY-STAT3 are then translocated to the nucleus. VI. The accumulated nuclear EGFR acts as a transcriptional co-activator, possibly in association with STAT3, to upregulate cyclin D1 expression, leading to the enhanced cancer cell proliferation and migration. VII. The pY291-Fas-dependent hyperactivity of STAT3 promotes expressions of its target genes, leading to the upregulation of Akt and Erk activities, and thus the enhanced cancer cell proliferation and migration.

Given increasing evidence of the non-apoptotic activities of Fas in tumor malignancies, it is important to understand the roles of Fas in the network of cancer-promoting pathways. Our data suggest that Fas in its phosphorylated pro-survival form is an essential signaling hub for the formation of a Fas/EGFR/STAT3 complex, which promotes the activation of the non-canonical signaling pathway of EGFR and STAT3, and thus the transmission of the growth factor signals directly from the plasma membrane to the tumor-promoting transcriptional targets in the nucleus via EGFR and STAT3 nuclear transport (Summarized in Fig. 8). Further investigation is needed to understand the exact function of phosphorylated Fas in the nucleus. Our identification of the novel cancer-promoting function of Fas in the upregulation of nuclear EGFR and STAT3 signaling along with our previous observation that Fas is upregulated in some types of tumors^13^ suggest that the pro-survival pY-Fas may be a valuable prognostic marker as well as a therapeutic target. The development of therapeutic strategies to target the Fas pro-survival signaling, such as by neutralizing the pro-survival soluble FasL^12^ and/or co-inhibition of Src family kinases and STAT3, may represent a rational approach for combinatory or sequential cancer therapies to delay disease recurrence and prolong disease-free survival.

## Methods

### Cell lines

Colon cell lines SW480 were purchased from the American Type Culture Collection (ATCC) and HCT116 from Leibniz-Institut, German collection of microorganisms and cell culture (DSMZ). All cells were maintained in RPMI 1640 + Glutamax I (Gibco) supplemented with 10% fetal bovine serum (FBS) and maintained at 37 °C, 5% CO_2_. For site-directed mutagenesis studies, stable cell lines were established by transducing cells with lentivirus vector (pLenti6) carrying C-terminally V5- or AcGFP-tagged wild-type Fas protein or Fas protein with Y291D or Y291F as previously described^13^.

### Antibodies and reagents

The sources of antibodies and reagents are as followed: anti-pY291 Fas (prepared as previously described^13^ and validated for immunoblotting^13^ and flow cytometry Fig. S8); anti-Src, anti-Yes (R&D Systems); anti-human Fas (C20, for immunoblotting) and anti-Cyclin D1 (Santa Cruz); anti-Fas (Apo1–3, for co-immunoprecipitation) (Enzo); anti-EGFR, anti-pY1068 EGFR, anti-pY705 STAT3, anti-STAT3, anti-pS473 Akt, anti-pT308 Akt, anti-Akt, anti-pErk1/2, and anti-Histone H3 (Cell Signaling Technology); anti-Erk1/2 (Sigma); anti-GAPDH (Calbiochem); horseradish peroxidase conjugated anti-rabbit and anti-mouse (Jackson ImmunoResearch); anti-mouse IgG-Alexa Flour 488 (Life Technology); Recombinant human EGF (Gibco), recombinant amphiregulin (R&D Systems); protein G sepharose beads (Zymed Laboratories); WST-1 (Interchim); Matrigel (BD Bioscience). Lipofectamine RNAiMAX reagent (Invitrogen) was used for siRNA transfection according to the manufacturer’s protocol for reverse transfection.

### siRNA

Src and Yes-1 siRNAs were from QIAGEN (catalog # SI02223921 and SI00302218, respectively). Control siRNA (Luciferase) (5′-CGUACGCGGAAUACUUCGA-3′) and STAT3 siRNA (5′-GGCGUCCAGUUCACUACUA-3′) and Fas siRNA (Exon 3: 5′-AAGGAGUACACAGACAAAGCC-3′, used for all experiments except those shown in Fig. 2F and Exon 9: 5′-GAAGCGUAUGACACAUUGA-3′, used for Fig. 2F) are from Eurofins.

### WST-1 Viability assay

Cells were seeded in RPMI + 10% FBS at 5 × 10^3^ cells/well in 96-well plate for 24 h. Cells were then washed with RPMI + 0.1% BSA and synchronized by serum deprivation in RPMI + 0.1% BSA for 24 h before treatment with an indicated reagent for 48 h. Following the treatment, the WST-1 reagent was added to each well. Cells were incubated for 4 h before measurement at 450 nm and 690 nm (reference) with a spectrometer (Biotek). After subtracting blank control, absorbance at 690 nm was subtracted from that obtained at 450 nm. The viability of the cells was calculated as a percentage of cell viability compared to control (untreated cells) using the following equation: % Viability = 100 × (A450–A690)_treated_/(A450–A690)_control_; A, absorbance.

### Boyden chamber cell migration assay

The chemotaxis migration assay using Boyden chambers was carried out as described^13^. Briefly, cells, pre-labeled with DilC18(3)-DS dye (Molecular Probes), were resuspended in RPMI medium supplemented with 0.1% fatty acid-free BSA (assay medium) at the concentration of 2 × 10^5^ cells/ml. Fluoroblok inserts (BD Bioscience) were placed in the wells of 24-well plates, each prefilled with 0.75 ml of assay medium without or with EGF. Cells (5 × 10^4^) were then added to the top chamber of the Fluoroblok membrane insert and allowed to migrate for 2 h at 37 °C, 5% CO_2_. Cells were then fixed with 4% paraformaldehyde and the fluorescence-labeled migrated cells on the underside of the inserts were imaged using a wide field inverted microscope with a 10x objective (Zeiss Axiovert). From 5 randomly taken images per insert, the number of cells migrated through the membrane pores was counted using the Analyze Particles function in FIJI software^48^.

### Three-dimensional cell culture in Alvetex scaffold

The Alvetex Scaffold discs in the 24-well plate format (Reinnervate) were prepared according to manufacturer’s instruction. Matrigel (0.8 mg/ml) was then applied to the discs and allowed to solidify to obtain the Matrigel-coated scaffold. Fifty µl of SW480 cell suspension (1.0 × 10^7^ cells/ml) was added to the center of each Alvetex®Scaffold disc. The plate was then incubated for 3 hours at 37 °C with 5% CO_2_ to allow the cells to settle into the scaffold. Then 1.5 ml of complete medium was added to each well. After 24 hours, the complete medium was replaced with 1 ml of fresh starving medium (RPMI + 0.1% BSA) and incubated for 24 hours. The starving medium was then replaced with 1.5 ml of the treatment medium without or with 0.01 ng/ml EGF. Cells were cultured at 37 °C with 5% CO_2_ with fresh treatment medium replacement every 2 days. After 10 days, the medium was removed and the discs were washed twice with PBS and fixed with 4% paraformaldehyde overnight. The discs were then washed with PBS, embedded in paraffin wax, sectioned to 10 µm thickness, and counterstained with hematoxylin and eosin. Brightfield micrographs of the sectioned discs were taken using a Zeiss Axioplan color microscope with 10X objective.

### SDS-PAGE and immunoblotting

Cells were seeded in 10-cm dishes at 4 × 10^6^ cells/dish or in 6-well plate at 2.5 × 10^5^ cells/well in RPMI + 10% FBS for 24 h. They were then washed with RPMI + 0.1% BSA and synchronized by serum deprivation in RPMI + 0.1% BSA for 24 h before treatment with EGF or AR. The detailed procedure for cell lysate collection and handling and SDS-PAGE and immunoblotting has been described^49^. Briefly, the medium was then removed and the lysis buffer (120 mM Tris-HCl pH 6.8, 4% SDS, 10 mM NaP-P, 10 mM NaF, 25 mM β-glycerol phosphate, 5 mM NaVO_4_, protease inhibitor cocktail), preheated at 95 °C (‘hot SDS’ buffer) was rapidly added to the cells. The lysate was collected and heated at 95 °C for 5 minutes. The lysate was stored at −80 °C until analyzed. The protein contents of the lysates were quantified using Dc protein assay (BioRad), and for each sample, 20 μg of protein was subjected to SDS-PAGE and immunoblotting. GAPDH served as a gel loading control except for Fig. 2B where Erk1/2 was used instead of GAPDH since the latter was affected by the suppression of Fas in HCT116 cells.

### Isolation of nuclei

One million cells were seeded in a 10-cm cell culture dish for 24 hours and subsequently synchronized by serum deprivation for 24 hours in RPMI + 0.1% BSA prior to an activation with indicated concentrations of EGF for an indicated time. Cell nuclei were isolated using the REAP procedure^50^. Briefly, cells were collected in 1 mL of PBS and centrifuged for 10 seconds at 10,000 rpm. Cells pellet were resuspended in 900 ul of cold PBS containing 0.1% NP-40 and centrifuged for 10 sec at 10,000 rpm. The supernatant was transferred to a new tube as cytoplasmic fraction. The pellet was washed by resuspension in 1 mL of PBS containing 0.1% NP-40 followed by centrifugation for 10 sec at 10,000 rpm. The washed pellet (nuclear fraction) was then resuspended in 100 µL of 1 × Laemmli buffer and sonicated briefly. The nuclear and cytoplasmic fractions were heated for 1 min at 95 °C and stored at −80 °C until further use. Prior to SDS-PAGE and immunoblotting, the protein contents of the fractions were quantified using Dc protein assay (BioRad) and, for each sample, an equal amount of protein was loaded on to the gel.

### Coimmunoprecipitation detected by immunoblotting

Fas coimmunoprecipitation was performed as previously described^13^. Briefly, the postnuclear supernatant (PNS) of cells was prepared by collecting cells in ice-cold lysis buffer, followed by sonication and subsequently centrifugation to remove cell debris. The PNS was then incubated with protein G agarose beads conjugated with anti-Fas (APO-1-3) at 4 °C for 18 h. The beads were washed and the immunoprecipitates were eluted from beads by heating in Laemmli buffer at 95 °C for 5 min, then subjected to SDS–PAGE, followed by immunoblotting.

### Immunoprecipitation detected by flow cytometry (IP-FCM)

IP-FCM performed according to the previously described^51^ with slight modifications. Briefly, polystyrene beads (Spherotech) were coated with a monoclonal antibody against Fas (Apo-1-3) as described by manufacturer’s instruction. To capture Fas complex in the nucleus, IP beads were incubated with lysate of the nuclear fraction of SW480 cells and washed to remove excess lysate. The washed IP beads were incubated with unrelated IgG or a primary antibody against Fas, pY1068 EGFR, or pY705 STAT3 followed by a corresponding secondary antibody. Then the IP beads were analyzed by flow cytometry.

### Flow cytometric analysis of phosphorylated proteins

Cells of interest were trypsinized and fixed in 4% paraformaldehyde at on ice for 20 minutes. Cells were washed and permeabilized in 0.3% saponin in PBS (permeabilization buffer) for 5 minutes. Cells were then washed with staining buffer (permeabilization buffer + 1% FBS) and incubated with a primary antibody for 1 h, washed, and incubated a corresponding secondary antibody for 30 min at room temperature. Cells were then washed and analyzed by flow cytometry.

## Electronic supplementary material

Supplementary figures

## Data Availability

The datasets generated during and/or analyzed during the current study are available from the corresponding author on reasonable request.
