# Supplementary material for: The tyrosine phosphorylated pro-survival form of Fas intensifies the EGF-induced signal in colorectal cancer cells through the nuclear EGFR/STAT3-mediated pathway
Source: Sci Rep. 2018 Aug 20;8:12424. doi: 10.1038/s41598-018-30804-z (PMC6102278; doi:10.1038/s41598-018-30804-z)
Supplement: Supplementary file 1 — Supplementary figures [file 41598_2018_30804_MOESM1_ESM.docx]

**The tyrosine phosphorylated prosurvival form of Fas intensifies the EGF-induced signal in colorectal cancer cells through the nuclear EGFR/STAT3-mediated pathway**

**Ly Ta Ngoc^1,2^, Krittalak Chakrabandhu^1, *^, Sébastien Huault^1^, and Anne-Odile Hueber^1, *^**

^1^ Université Côte d’Azur, CNRS, Inserm, iBV, France.

^2^ The University of Da-Nang, University of Science and Technology, Viet Nam

**Supplementary Figures and Information**

**
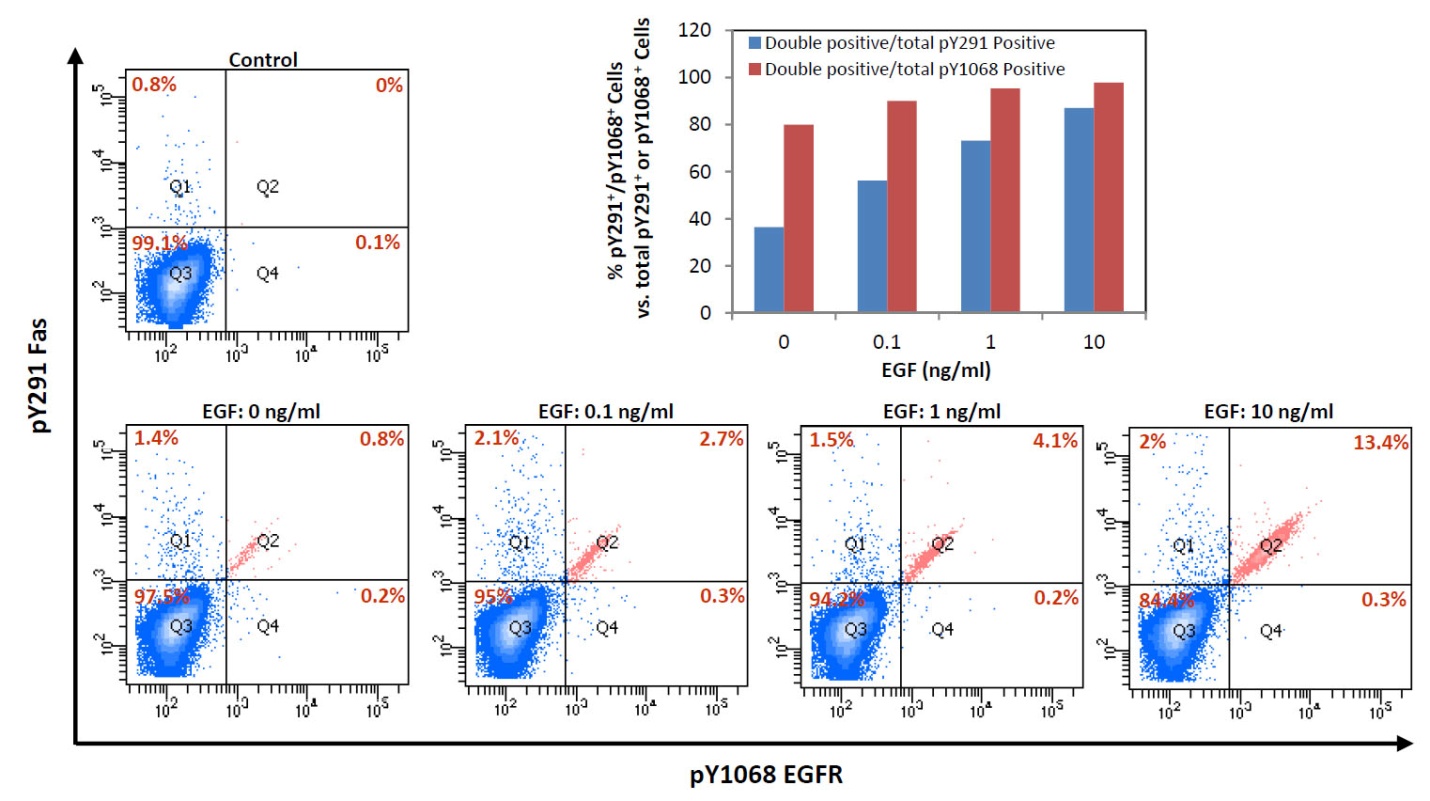
**

**Fig. S1. pY291 Fas level rises concurrently with pY1068 EGFR upon cell treatment with EGF**

SW480 cells were synchronized to the G1 phase by serum starvation for 24h before being treated with the indicated concentration of EGF (ng/ml) for 5 minutes. Cells were then processed for flow cytometric analysis of phosphorylated proteins as described in Methods section. The plot on the upper right shows the percentage of cells that were positive for both pY291 Fas and pY1068 EGFR compared to all cells that were positive for pY291 Fas (blue) or compared to all cells that were positive for pY1068 EGFR (red). Note that the majority of cells that were pY1068 EGFR-positive were also pY291 Fas-positive.

**
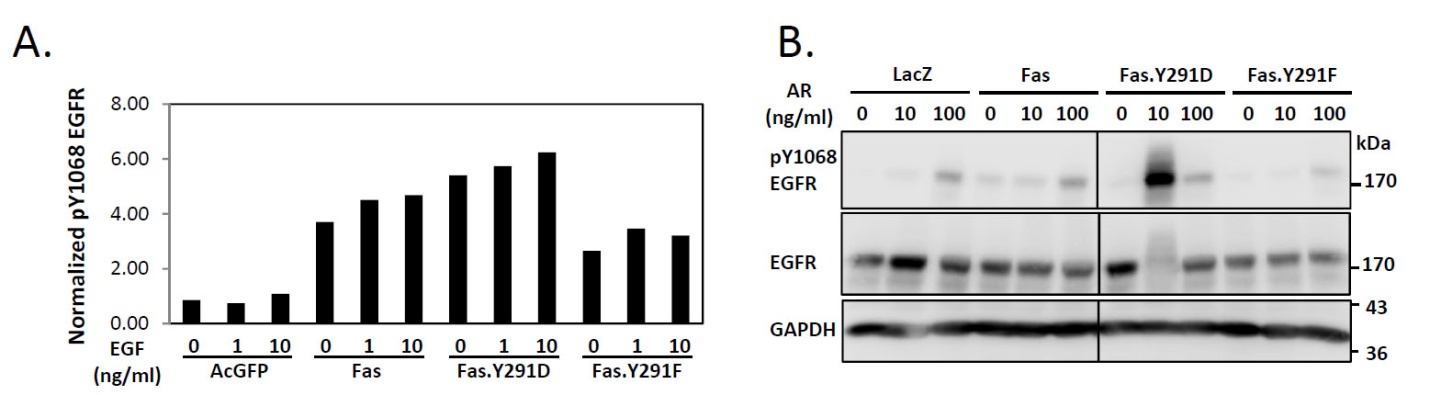
**

**Fig. S2. The prosurvival form of Fas promotes ligand-induced activation of EGFR**

(A). SW480 cell lines stably expressing AcGFP or indicated AcGFP-tagged protein were synchronized to the G1 phase by serum deprivation for 24h and then incubated with an indicated concentration of EGF for 5 minutes. Cells were then processed for flow cytometric analysis of phosphorylated proteins as described in Methods section. Data are presented as the median fluorescent intensity (MFI) of pY1068 EGFR staining normalized by MFI of AcGFP which was the measure of the expression level of each indicated AcGFP-tagged Fas protein (AcGFP). Data from cells with equivalent levels of AcGFP expression are shown. Data are representative of 2 independent experiments. (B). SW480 cell lines stably expressing control protein (LacZ.V5), V5-tagged wild-type Fas, the pY291 Fas proxy (Fas.Y291D), or unphosphorylated Y291 Fas (Fas.Y291F) were synchronized for 24h then treated with indicated dose of amphiregulin (AR) for 5 minutes before cell lysates were collected and subjected to SDS-PAGE and immunoblotting with indicated antibodies. Data are representative of 2 independent experiments.

**
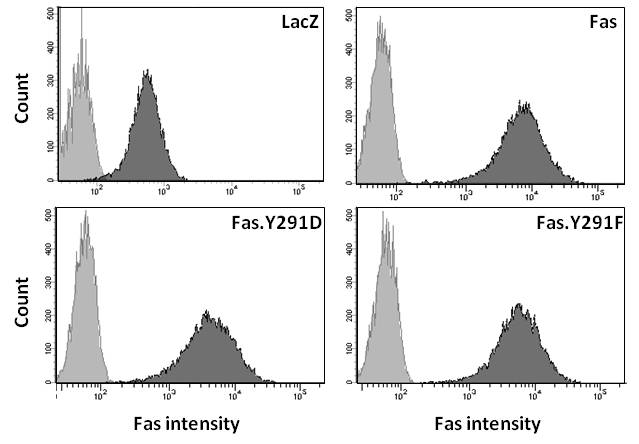
**

**Fig. S3. Expression of Fas in stable cell lines used in site-directed mutagenesis studies.**

Flow cytometric analysis showing equivalent levels of Fas surface expression of SW480 cells stably over-expressing indicated V5-tagged proteins (A) (grey, isotype control; black, anti-Fas antibody).


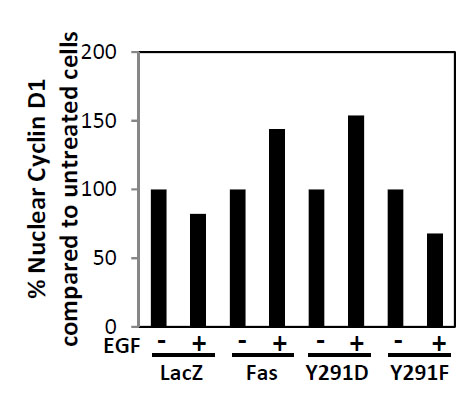


**Fig. S4. The prosurvival form of Fas is necessary for the EGF-induced upregulation of Cyclin D1 in the nucleus.**

SW480 cell lines stably expressing control protein (LacZ.V5), V5-tagged wild-type Fas, the pY291 Fas proxy (Fas.Y291D), or unphosphorylated Y291 Fas (Fas.Y291F) were synchronized for 24h then incubated without or with 10 ng/ml EGF for 3h. Cell nuclei were then isolated as described in the Methods section. The isolated nuclei were stained with anti-Cyclin D1 antibody followed by a corresponding secondary antibody. Subsequently, they were counter-stained with anti-tubulin antibody followed by a corresponding secondary antibody. Control cells were stained with the secondary antibody alone. The nuclei were analyzed by flow cytometry. Gating was used to exclude events that were positive for tubulin, which is a marker for cytoplasmic content. Data are presented as the percentage of median fluorescent intensity (MFI) of Cyclin D1 staining compared to untreated cells. Data are representative of 2 independent experiments.


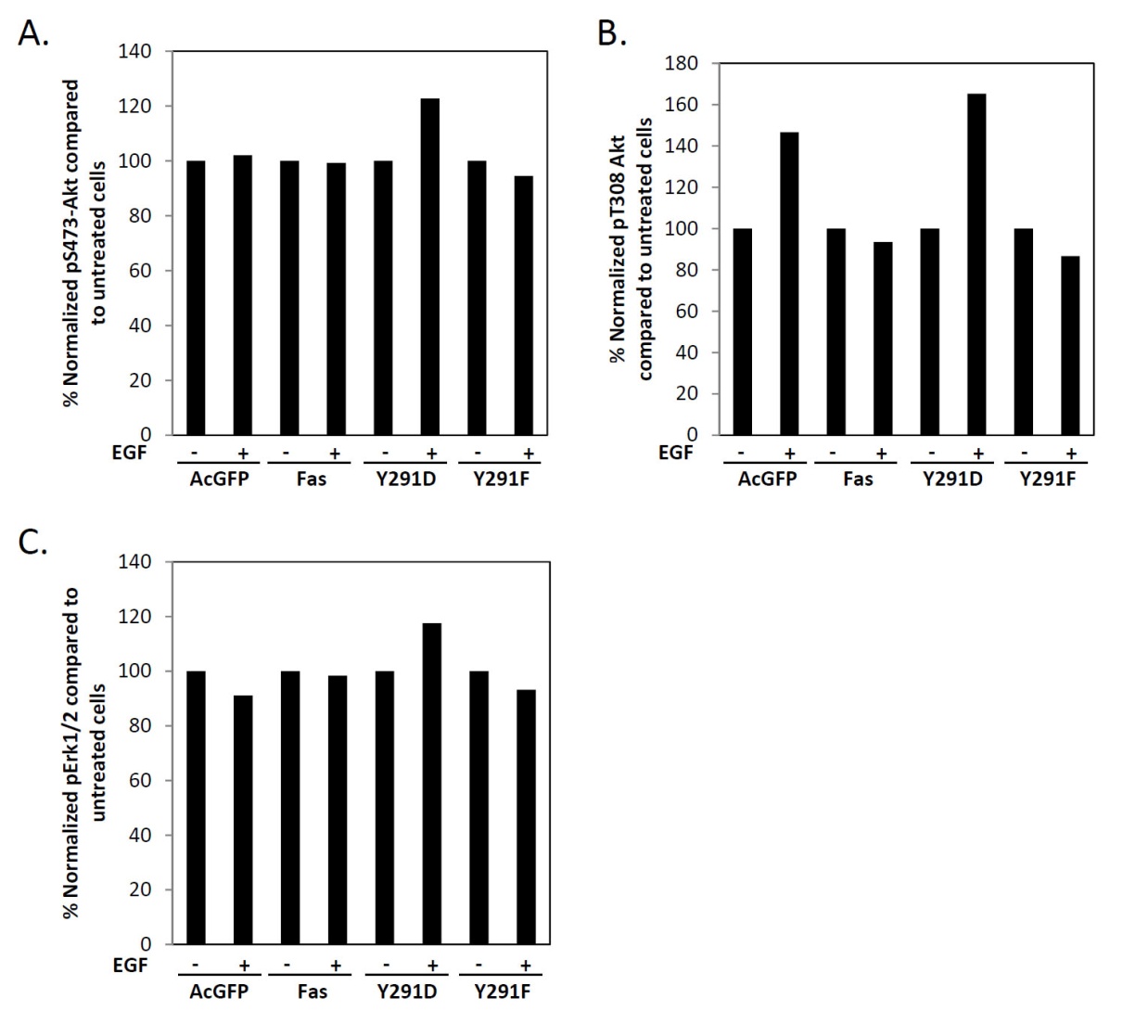


**Fig. S5. EGF-induced activation of Akt and Erk pathway requires the prosurvival form of Fas.**

SW480 cell lines stably expressing AcGFP or indicated AcGFP-tagged protein were synchronized to the G1 phase by serum deprivation for 24h and then incubated with 10 ng/ml EGF or without (control) for 5 minutes. The levels of pS483 Akt (A), pT308 Akt (B), and pErk1/2 (C) were then analyzed by flow cytometry. The median fluorescent intensity (MFI) of the phospho-protein staining was normalized by MFI of AcGFP which was the measure of the expression level of each indicated AcGFP-tagged Fas protein (AcGFP). Data are presented as the percentage of normalized MFI of the phospho-protein staining compared to untreated cells. Data from cells with equivalent levels of AcGFP expression are shown. Data are representative of 2 independent experiments.

**
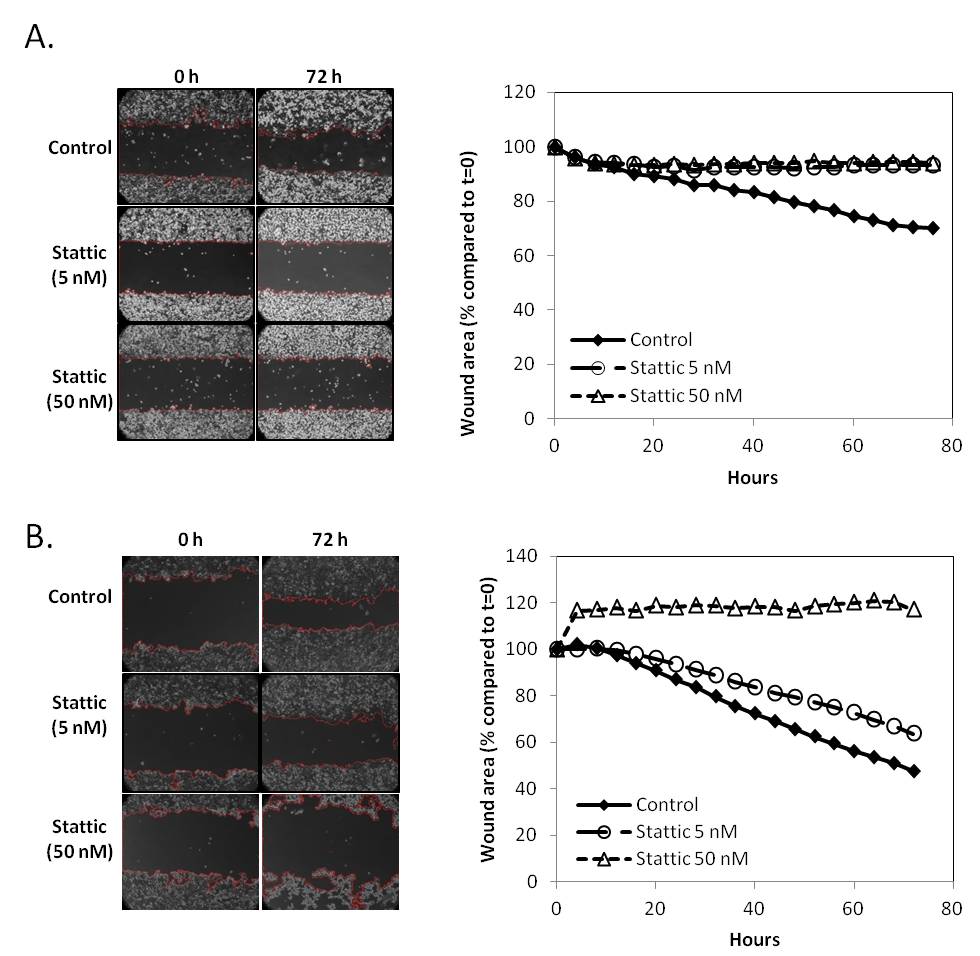
Fig. S6. Colorectal cancer cell migration is inhibited by Stattic, an inhibitor of STAT3 activation and dimerization**

SW480 (A) and HCT116 (B) cells were subjected to wound healing assay in the presence of indicated concentrations of Stattic, an inhibitor of STAT3 activation and dimerization^1^. Timelapse images of the cells were taken every 4 hours for 72 hours. The images of the cells at 0 h and 72 h are shown (A and B, left panels). The wound area was quantified for each time point and presented as the percentage of wound area at each time point compared to the initial time point (t=0 h) (A and B, right panel).

**
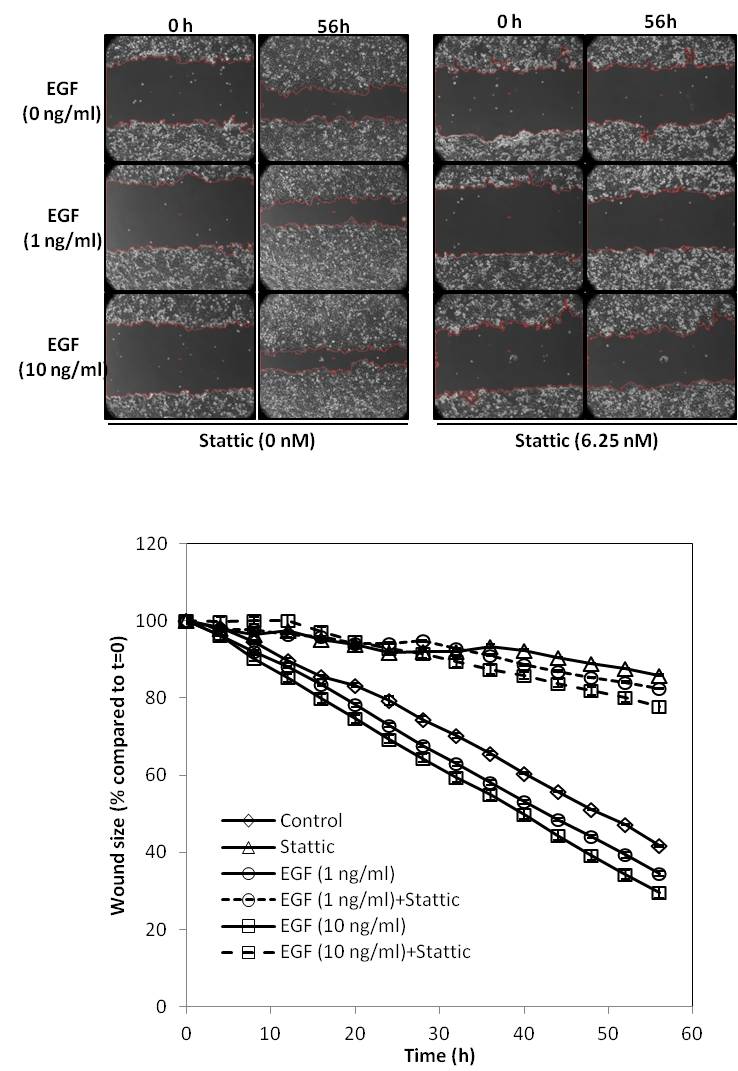
**

**Fig. S7. Inhibition of STAT3 suppressed EGF-induced colorectal cancer cell migration**

SW480 cells were subjected to wound healing assay in the presence of indicated concentrations of EGF and 6.25 nM Stattic. Timelapse images of the cells were taken every 4 hours for 56 hours. The images of the cells at 0 h and 56 h are shown (top panel). The wound area was quantified for each time point and presented as the percentage of wound area at each time point was calculated as in Fig. S2.


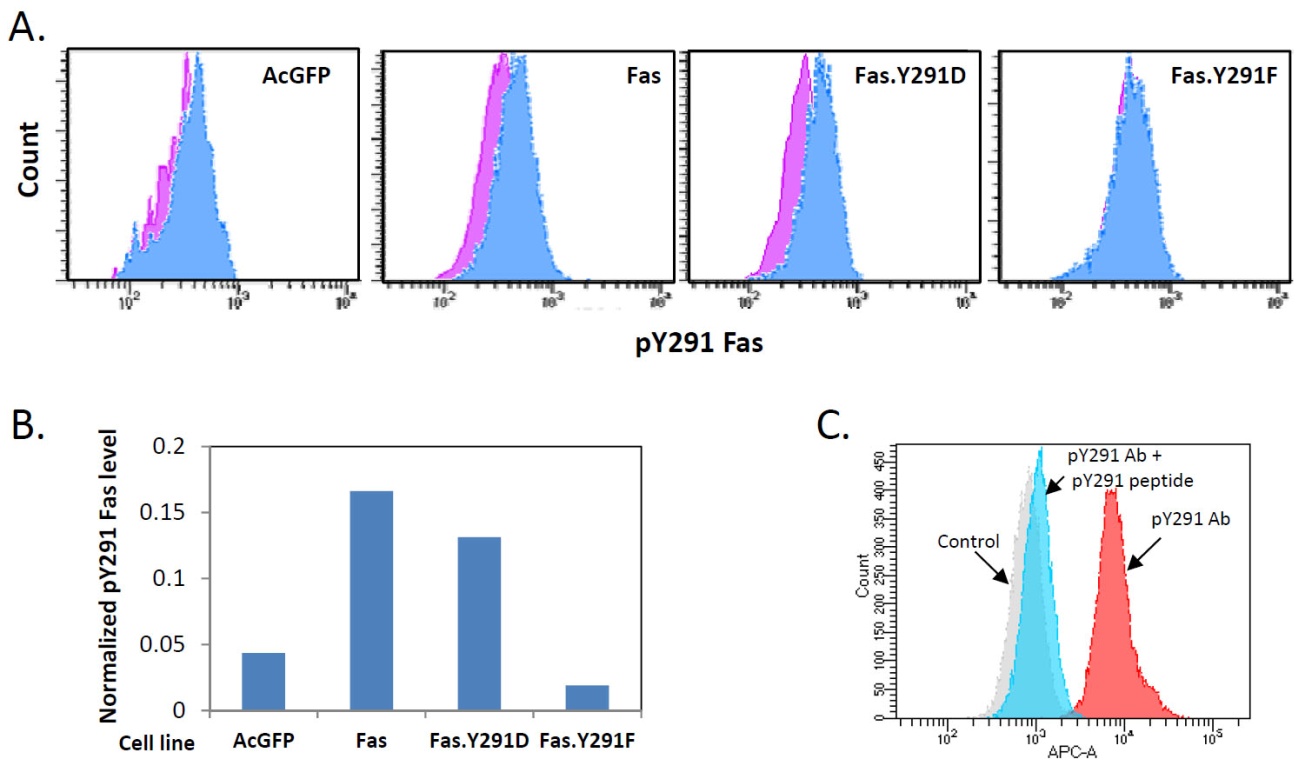


**Fig. S8. Validation of pY291 Fas antibody for flow cytometric application**

(A). SW480 cell lines stably expressing AcGFP or indicated AcGFP-tagged protein were processed for flow cytometric analysis of phosphorylated proteins as described in Methods section. Cells were incubated with control IgG or with the antibody against pY291 Fas (33A9.2) followed by secondary antibody and analyzed by flow cytometry. Data from cells with equivalent levels of AcGFP expression are shown. Pink, cells incubated with control IgG; blue, with anti-pY291 Fas antibody. (B). From cells prepared and analyzed as in (A), the median fluorescent intensity (MFI) of the pY291 Fas staining was normalized by MFI of AcGFP, which was the measure of the expression level of each indicated AcGFP-tagged Fas protein (AcGFP). Note the lack of pY291 Fas staining in cells expressing dephosphorylate Fas mimetic (Fas.Y291F) in (A) and (B). (C). SW480 cells were processed for pY291 Fas measurement by flow cytometry as in (A). Grey, cells stained with secondary antibody alone; blue, cells incubated with anti-pY291 Fas in the presence of 10000x molar excess of pY291 peptide to specifically block the antibody binding to pY291 Fas; red, cells incubated with anti-pY291 Fas antibody in the absence of the blocking peptide. Note the inhibition of anti-pY291 Fas antibody staining in the presence of the pY291 Fas blocking peptide.

**Fig. S9. Full blot images**

**
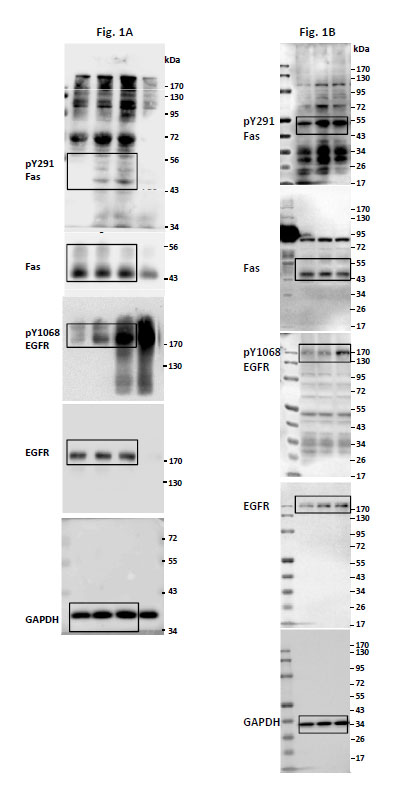
**

**
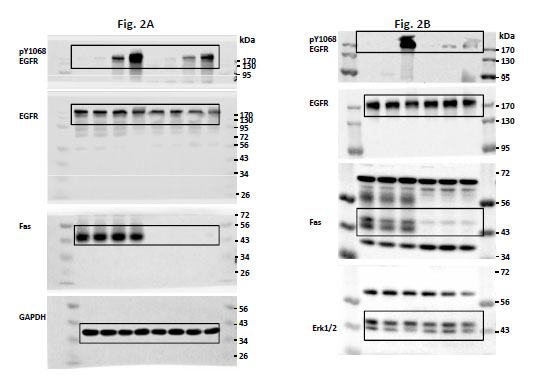
**

**
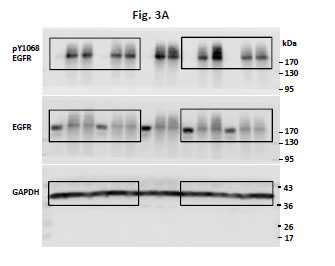
**

**
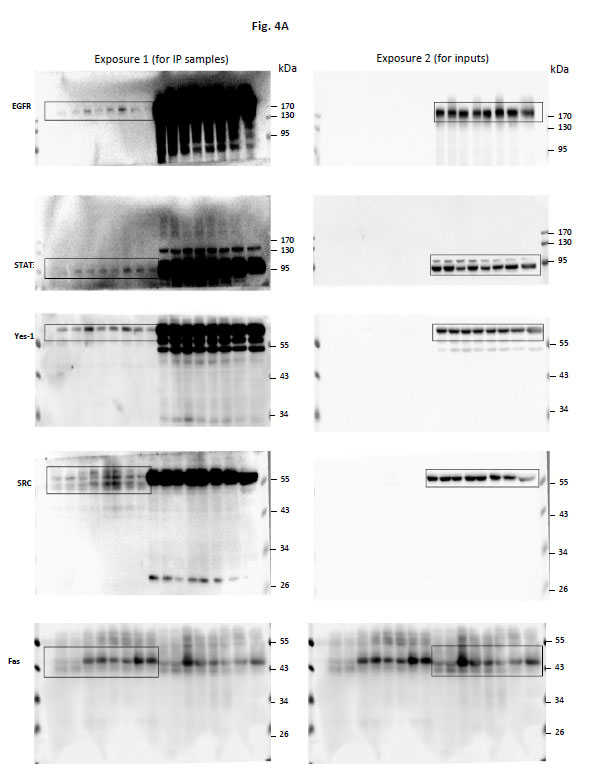
**

**
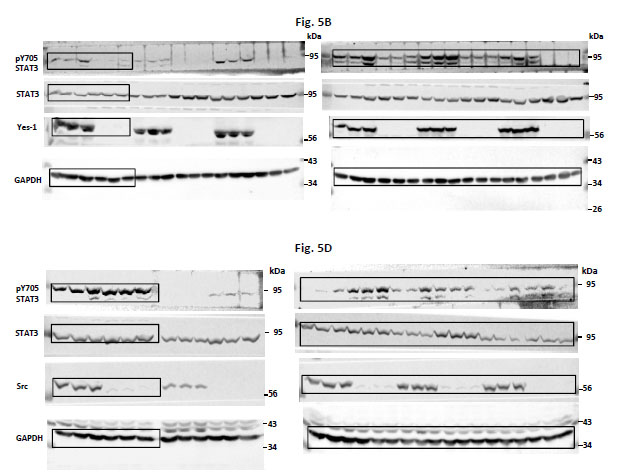
**

**
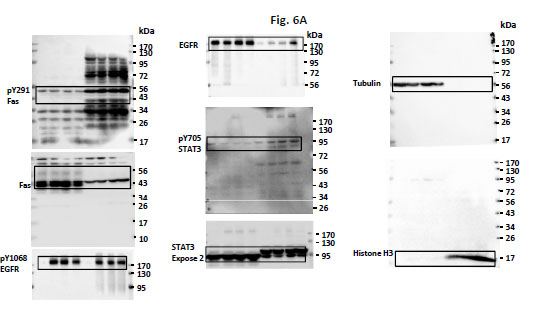
**

**
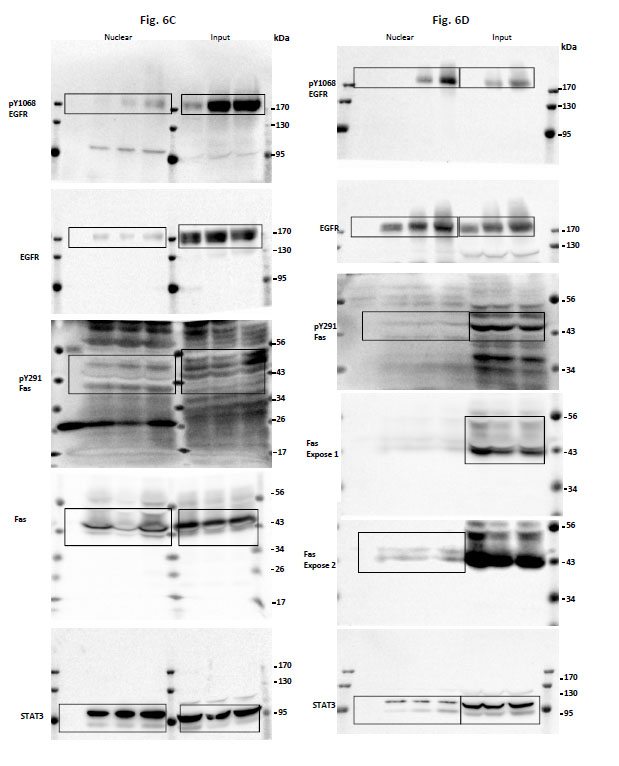
**

**
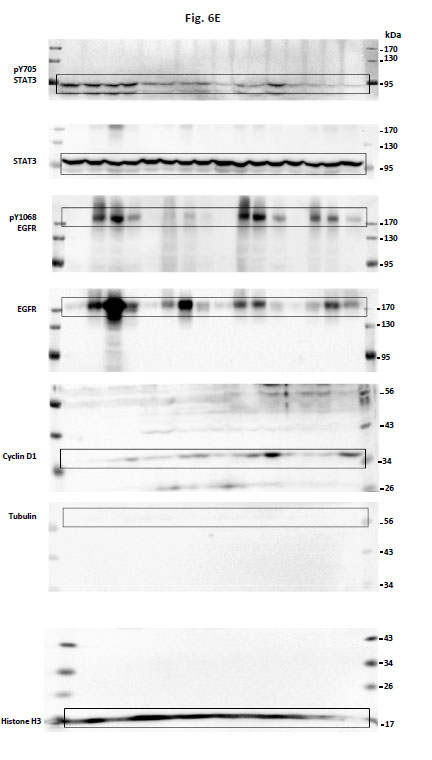
**

**
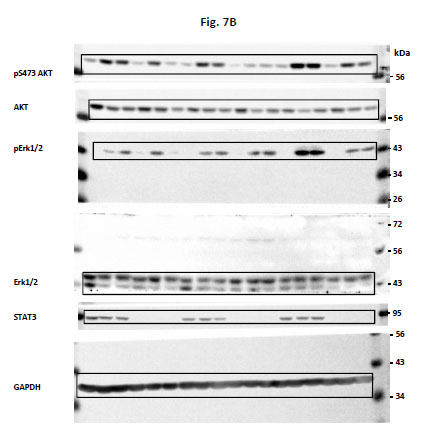
**

**Supplementary methods**

**Cell surface Fas expression analysis by flow cytometry**

Cells were seeded in 6-well plate at 5x10^5^ cells/well in RPMI+10% FBS for 24h. After trypsinization and a wash with PBS, cells were placed on ice and stained with a PE-conjugated antibody against the extracellular domain of Fas (DX2, Miltenyi). Cells were then subjected to flow cytometric analysis (LSRFortessa, BD Biosciences) and data were analyzed using FACSDIVA software (BD Biosciences).

**Wound healing assay**

HCT116 cells were grown to 90% confluence in 12-well plate. A uniform wound was created on the cell monolayer using a 1-mm wide plastic strip. Cells were then rinsed twice with serum-free culture media to remove floating cells. Medium containing 10% FBS with EGF and/or Stattic (Calbiochem) according to the experiment was added to the cells. Timelapse images of the cells were taken with a phase-contrast microscope equipped with temperature/CO2-controlled chamber. The wound closure images were captured for 72 hours with 4 hours interval. The wound area was quantified for each time point using FIJI software^2^ (ImageJ). The percent of wound closure was calculated as the wound area at each time point compared to the initial wound area.

**Supplementary references:**

1 Schust, J., Sperl, B., Hollis, A., Mayer, T. U. & Berg, T. Stattic: a small-molecule inhibitor of STAT3 activation and dimerization. *Chem Biol* **13**, 1235-1242 (2006).

2 Schindelin, J. *et al.* Fiji: an open-source platform for biological-image analysis. *Nat Methods* **9**, 676-682 (2012).
